# Complementing 16S rRNA Gene Amplicon Sequencing with Total Bacterial Load To Infer Absolute Species Concentrations in the Vaginal Microbiome

**DOI:** 10.1128/mSystems.00777-19

**Published:** 2020-04-07

**Authors:** Florencia A. Tettamanti Boshier, Sujatha Srinivasan, Anthony Lopez, Noah G. Hoffman, Sean Proll, David N. Fredricks, Joshua T. Schiffer

**Affiliations:** a Vaccine and Infectious Disease Division, Fred Hutchinson Cancer Research Center, Seattle, Washington, USA; b Department of Laboratory Medicine, University of Washington, Seattle, Washington, USA; c Department of Biostatistics, University of Washington, Seattle, Washington, USA; d Clinical Research Division, Fred Hutchinson Cancer Research Center, Seattle, Washington, USA; e Department of Medicine, University of Washington, Seattle, Washington, USA; f Department of Microbiology, University of Washington, Seattle, Washington, USA; Northern Arizona University

**Keywords:** applied microbiology, gene amplicon sequencing, quantitative PCR, vaginal microbiome

## Abstract

Whereas 16S rRNA gene amplicon sequencing quantifies relative abundances of bacterial taxa, variation in total bacterial load between samples restricts its ability to reflect absolute concentrations of individual bacterial species. Quantitative PCR (qPCR) can quantify individual species, but it is not practical to develop a suite of qPCR assays for every bacterium present in a diverse sample. We sought to determine the accuracy of an inferred measure of bacterial concentration using total bacterial load and relative abundance. We analyzed 1,320 samples from 20 women with a history of frequent bacterial vaginosis who self-collected vaginal swabs daily over 60 days. We inferred bacterial concentrations by taking the product of species relative abundance (assessed by 16S rRNA gene amplicon sequencing) and bacterial load (measured by broad-range 16S rRNA gene qPCR). Log_10_-converted inferred concentrations correlated with targeted qPCR (*r* = 0. 935, *P* < 2.2e–16) for seven key bacterial species. The mean inferred concentration error varied across bacteria, with rarer bacteria associated with larger errors. A total of 92% of the >0.5-log_10_ errors occurred when the relative abundance was <10%. Many errors occurred during early bacterial expansion from or late contraction to low abundance. When the relative abundance of a species is >10%, inferred concentrations are reliable proxies for targeted qPCR in the vaginal microbiome. However, targeted qPCR is required to capture bacteria at low relative abundance and is preferable for characterizing growth and decay kinetics of single species.

**IMPORTANCE** Microbiome studies primarily use 16S rRNA gene amplicon sequencing to assess the relative abundance of bacterial taxa in a community. However, these measurements do not accurately reflect absolute taxon concentrations. We sought to determine whether the product of species’ relative abundance and total bacterial load measured by broad-range qPCR is an accurate proxy for individual species’ concentrations, as measured by taxon-specific qPCR assays. Overall, the inferred bacterial concentrations were a reasonable proxy of species-specific qPCR values, particularly when bacteria are present at a higher relative abundance. This approach offers an opportunity to assess the concentrations of bacterial species and how they change in a community over time without developing individual qPCR assays for each taxon.

## Abstract (precis)

Microbiome studies primarily use 16S rRNA gene amplicon sequencing to assess the relative abundance of bacterial taxa in a community. However, these measurements do not accurately reflect absolute taxon concentrations. We sought to determine whether the product of species’ relative abundance and total bacterial load measured by broad-range qPCR is an accurate proxy for individual species’ concentrations, as measured by taxon-specific qPCR assays. Overall, the inferred bacterial concentrations were a reasonable proxy of species-specific qPCR values, particularly when bacteria are present at a higher relative abundance. This approach offers an opportunity to assess the concentrations of bacterial species and how they change in a community over time without developing individual qPCR assays for each taxon.

## INTRODUCTION

For most infectious diseases, the absolute concentration of a single pathogen is often the most specific marker of disease severity and therapeutic response (1–3). In contrast, studies of bacterial communities usually rely on broad-range consensus sequence PCR of taxonomically informative genes (such as 16S rRNA) coupled with next-generation sequencing (NGS) to assess relative, but not absolute abundances of bacteria. At a mechanistic level, specific combinations of bacteria and bacterial gene products are thought to play a causative role in the pathogenesis of many microbiome associated conditions (4–6), and this approach of characterizing the microbiota is valuable. However, the absolute concentrations of individual bacterial taxa within communities may be a better predictor of biological activity or disease risk compared to relative abundances of these taxa. Quantitating absolute concentrations of individual species with qPCR is time intensive, requires the generation of a standard curve for each organism using known concentrations of DNA, is expensive, and is only available in specialized laboratories. Moreover, each qPCR assay requires significant development and validation costs. qPCR is therefore not typically comprehensive for all species in a community. In addition, selection of the most appropriate species for analysis may reflect investigator bias.

A method to infer absolute concentration of multiple bacterial species from NGS data would be extremely useful for the field, including studies of the vaginal microbiome. NGS amplicon sequencing is a fractional approach that has been used to help define conditions such as bacterial vaginosis (7–10) and to identify enhanced risk for other sexually transmitted infections and preterm delivery (11, 12). However, total bacterial load may vary significantly between and within individuals over time even over the course of a single day (8). Therefore, relative abundances may not accurately represent absolute concentrations. Consequently, as shown recently in the gut microbiome, relative abundances may identify spurious disease associations which may in fact be driven by total microbial load (13).

Here, we demonstrate that multiplying relative abundance data (composition) by estimates of total bacterial DNA as measured by a broad-range 16S rRNA gene qPCR assay provides useful estimates of absolute concentrations of bacterial DNA for a given species in a sample. This technique has already been used in studies of the penile microbiome, though without formal validation (14), and in the fecal microbiome with limited validation (15). Here, we validate inferred concentrations by comparison of absolute concentrations measured by targeted qPCR assay for seven key species in the vaginal microbiome. We found that whereas inferred concentrations are accurate for most samples, they are prone to error when relative abundance is low and may misrepresent kinetics of individual species during critical periods of expansion from low bacterial abundance and during clearance.

## RESULTS

### Comparison of longitudinal profiles highlight differences between relative abundance and absolute concentration measurements.

**FIG 1:**
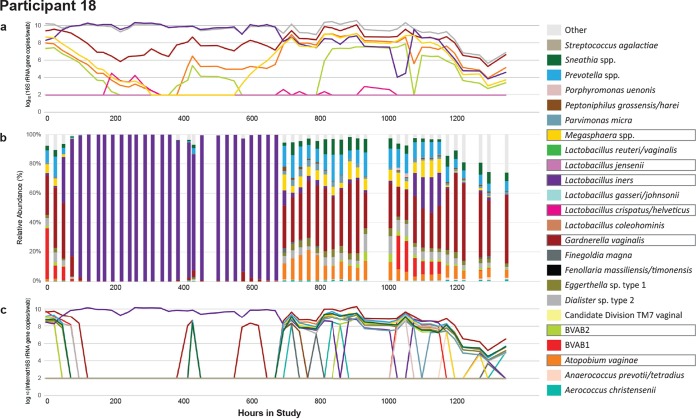
Complex bacterial kinetics in the vaginal niche in a representative study participant. Daily samples from a woman, participant 18, who performed self-swabbing of the vagina were analyzed by targeted qPCR of seven specific species (a), high-throughput sequencing using 16S rRNA (b), and inferred concentration for species with a relative abundance above 1% (c). Boxes around taxa indicate they were measured using qPCR. qPCR allows measures of the absolute concentration, whereas broad-range PCR with sequencing provides a measure of the bacterial diversity in a given sample. Targeted qPCR often detects shifts in single species prior to NGS. Inferred concentration follows qPCR more closely than does the relative abundance and may project the concentration of species for which targeted qPCR assays are not available. Traces for the remaining participants can be found in Fig. S1.

We compared absolute concentration and relative abundance from the same samples measured within individuals over the course of the study. The bacterial kinetics observed for a single participant are shown in Fig. 1a and b. The individual shown underwent dynamic changes in bacterial profile with notable shifts between low to high diversity states. The bacterial kinetics of the other 19 participants can be found in Fig. S1 in the supplemental material.

10.1128/mSystems.00777-19.1 FIG S1 Vaginal microbiome profile for all study participants. Participants 08 and 09 show 8 hourly samples, remaining subjects show daily morning samples. (a) Targeted qPCR of seven bacterial species; (b) high-throughput amplicon sequencing using 16S rRNA gene for top 24 species across entire data set; (c) inferred concentration for bacterial species with relative abundances above 1%. Boxes around taxa indicate they were measured using qPCR. qPCR allows measures of absolute concentration, whereas broad range PCR with sequencing provides a measure of bacterial diversity in a given sample. Targeted qPCR often detects shifts in single species prior to NGS. Inferred concentration follows qPCR more closely than relative abundance does and may project concentration of species for which targeted qPCR assays are not available. Download FIG S1, PDF file, 2.6 MB. Copyright © 2020 Tettamanti Boshier et al. 2020 Tettamanti Boshier et al. This content is distributed under the terms of the Creative Commons Attribution 4.0 International license.

In five of the participants, shifts in composition appear less abruptly when measured by single-species qPCR than by NGS (Fig. S1). For example, for the participant shown in Fig. 1, the absolute concentration of *A. vaginae* increases on day 17 (h 415), but its relative abundance does not show a consistent increase until day 28 (h 671), although there are some nonzero abundances in 4/9 samples before this point. From day 0 to day 7 (h 168), the participant received metronidazole for bacterial vaginosis (BV): qPCR shows an exponential decline in BV-associated species absolute concentrations in accordance with previous studies (16); yet, NGS shows a much more abrupt shift toward Lactobacillus iners predominance. NGS can also fail to capture low-level colonization of bacteria, such as that of Gardnerella vaginalis on days 6 to 11 (h 150 and 261). Several high-diversity samples have highly prevalent species which were not measured with qPCR in this study, such as Prevotella bivia, from day 28 onward (h 671). As previously noted, high diversity states are often concurrent with high absolute concentrations of Gardnerella vaginalis, Atopobium vaginae, BVAB2, and *Megasphaera*, which all have been associated with BV (8, 10, 17). These observations, which can be made for many of the individuals in this cohort, highlight that qPCR provides more granular estimates for measuring single species kinetics, while NGS is optimal to estimate bacterial diversity in high diversity communities.

**FIG 2:**
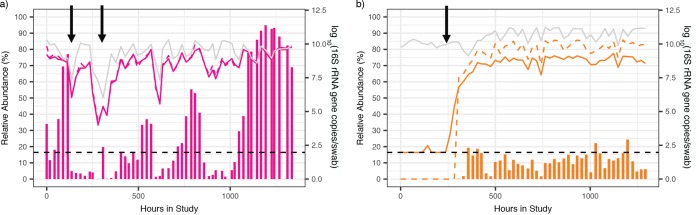
Relative abundance estimates can misrepresent actual concentrations due to shifts in total bacterial load. Examples of species-specific profiles in two participants for two different species L. crispatus, participant 06 (a), and *Megasphaera*, participant 17 (b). Vertical bars show the relative abundance (%, left *y* axis), solid lines indicate the absolute concentrations measured by qPCR, the gray line indicates the total bacterial load, and the dashed lines indicate inferred concentrations (all right *y* axis). The dashed black line indicates detection threshold for qPCR data (93.8 16S rRNA gene copies per swab). Arrows indicate time points when the relative abundance changes are discordant from the absolute concentration changes, which often occur when bacterial loads shift dramatically or when the relative abundance is low. Examples for the remaining species can be found in Fig. S2.

We next focused on comparing relative abundance and absolute concentration for individual species’ kinetics. Examples for two species, L. crispatus and *Megasphaera* sp., are shown in Fig. 2 (examples for the remaining five species are in Fig. S2 in the supplemental material). There were time points at which the absolute and relative abundance measures demonstrated opposing or differing kinetics, often due to concurrent large shifts in the total bacterial load or single species abundance. These are indicated by arrows in Fig. 2. Thus, the relative abundance may misrepresent the absolute concentration when not accounting for total bacterial load. Together, these observations identify a potential role for inferred concentrations, which can be calculated for all bacterial species present in the sample by NGS, when characterizing the microbiota.

10.1128/mSystems.00777-19.2 FIG S2 Relative abundances estimates can misrepresent actual concentrations due to shifts in total bacterial load. Vertical bars show relative abundance (%, left *y* axis), solid lines are absolute concentrations measured by qPCR, and the gray line is total bacterial load (both right *y* axis). The dashed black line indicates detection threshold for qPCR data (93.8 16S rRNA gene copies per swab). Arrows indicate obvious time points when relative abundance changes are discordant from absolute abundance changes, often when bacterial loads shift dramatically, or relative abundance is low. All seven species measured with qPCR in the study are included in separate panels from individual participants. (a) L. jensenii, participant 20; (b) *L. iners*, participant 02; (c) *G. vaginalis*, participant 18; (d) BVAB2 participant 15; (e) *A. vaginae*, participant 18. Download FIG S2, PDF file, 1.9 MB. Copyright © 2020 Tettamanti Boshier et al. 2020 Tettamanti Boshier et al. This content is distributed under the terms of the Creative Commons Attribution 4.0 International license.

### Noise detection analysis indicates limited impact of sampling variance in observed dynamics.

We next sought to assess whether observed shifts in qPCR values could be a result of noise related to sampling or laboratory variability rather than true longitudinal shifts in abundance. We used detection theory to estimate the sampling noise from our longitudinal data of absolute concentration. This technique decomposes the data into two parts: a signal (true concentration in a sample) and noise, the source of which may be biological, technical, sampling, or any combination of these. Further details of the technique can be found in the methods section.

**FIG 3:**
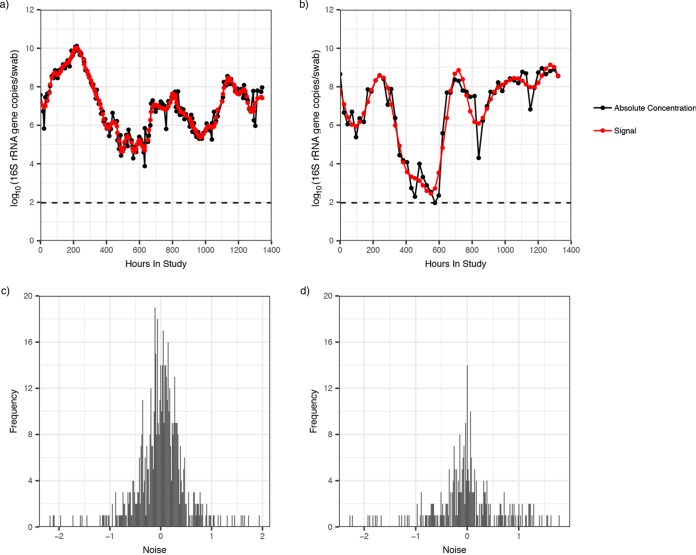
Noise detection analysis indicates small sampling variance. The signal and noise decomposition of two longitudinal profiles is shown. Measurements from targeted qPCR assays are indicated in black, signal detected by 25% low-pass filter is indicated in red for *L. iners* (a) and BVAB2 (b) in separate participants. In both cases, the signal closely matches the absolute concentration measurements. Consequently, the noise detected is of very low amplitude. The distribution of noise across all 20 participants is shown for *L. iners* (c) and BVAB2 (d). The noise distribution is low for both species, though a narrower range of noises is reported for *L. iners* than for BVAB2. The noise for both measurements has a mean of zero and a variance of 0.19 and 0.41 log_10_(16S rRNA gene copies per swab), respectively.

In Fig. 3a and b, we show the longitudinal profile of *L. iners* and BVAB2 for two participants. We found that the detected signal (shown in red) closely follows the measured data (shown in black), with only slight deviations. The same trend was identified in all other participants, and for each species. In Fig. 3c and d, we show the distribution of the detected noise for *L. iners* and BVAB2 across all participants. The detected noise had a mean of zero and a small variance [0.19 and 0.41 log_10_(16S rRNA gene copies per swab), respectively]. The same was found for the total bacterial load and all other species (Fig. S3).

10.1128/mSystems.00777-19.3 FIG S3 Noise detection analysis suggests limited impact of sampling variance. Distribution of noise across all 20 participants for total bacterial load (a), L. crispatus (b), L. jensenii (c), *G. vaginalis* (d), *Megasphaera* (e), and *A. vaginae* (f). The noise is 0 log_10_(16S rRNA gene copies/swab) for all measurements. Download FIG S3, PDF file, 0.3 MB. Copyright © 2020 Tettamanti Boshier et al. 2020 Tettamanti Boshier et al. This content is distributed under the terms of the Creative Commons Attribution 4.0 International license.

In our study, species are observed to undergo a change of up to 8.2 log_10_, and the total bacterial load can change 4.9 log_10_ over 60 days. These observed changes are much greater than the noise being estimated by our technique, which suggests that the dynamics being captured are most likely biological rather than noise.

### Inferred concentrations are predictive of absolute concentrations measured by qPCR.

For each species we calculated inferred concentrations by multiplying total bacterial load by NGS-relative abundance, as shown in equation 1. We then compared these with absolute concentration as measured by targeted qPCR assay for the seven key species. For each species, inferred bacterial concentration closely tracked absolute concentration for most samples (Fig. 1c; see also the dotted line in Fig. 2 and Fig. S2). In many instances and for most species, there were no obvious extreme discordance noted (Fig. 2a and Fig. S2). For some species, however, such as *Megasphaera* and BVAB2, inferred concentration consistently overestimated the absolute concentration by an order of magnitude (Fig. 2b and Fig. S2d). In a subset of samples, for all species, inferred concentration was zero while qPCR levels were positive, leading to profound discordance between the inferred and absolute concentrations: this was most often noted at low absolute concentration (Fig. 2).

**FIG 4:**
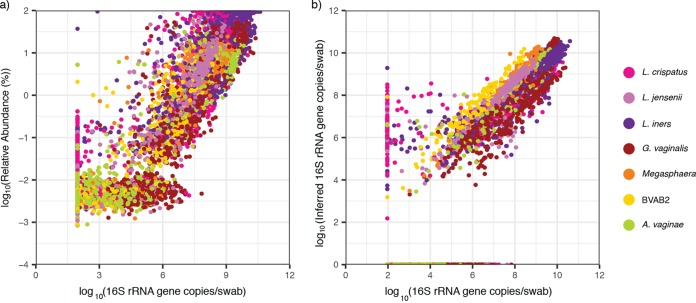
The inferred concentration slightly improves correlation with the absolute concentration compared to the relative abundance. (a) Scatterplot of the absolute concentration versus the relative abundance. Pearson correlation coefficient (PCC): *r* = 0.932 and *P* < 2.2e–16. (b) Scatterplot of the absolute concentration versus the inferred concentration. PCC: *r* is 0.935 and *P* < 2.2e–16. Both axes are plotted on a logarithmic scale. Samples which were determined to be negative by NGS but not by targeted qPCR are plotted on the *x* axis, while samples negative by targeted qPCR but determined to be positive by NGS are listed on the reported threshold for targeted qPCR (93.8 16S rRNA gene copies per swab). The relative abundances and inferred concentrations were generally falsely negative at low absolute concentrations. Variance in the relationship between the absolute concentration and the relative abundance is inversely proportional to species concentrations (Breusch-Pagan test, *P* = 2e–3), whereas this relationship was not statistically significant between the absolute concentration and the inferred abundance (Breusch-Pagan test, *P* = 0.06).

We compared the correlation between the relative abundance and the absolute concentration (*r* = 0.932, *P* < 2.2e–16; Fig. 4a) to the correlation between the inferred concentration and the absolute concentration (*r* = 0.935, *P* < 2.2e–16; Fig. 4b). The two correlation coefficients are not statistically different (Hittner test, *P* > 0.08) (18). Species-specific correlations are noted. For the inferred concentrations, *Megasphaera* and BVAB2 produced the strongest correlation, followed by L. crispatus, *A. vaginae*, and L. jensenii; *G. vaginalis* and *L. iners*, which are often present at moderate concentrations (∼10^6^ 16S rRNA gene copies per swab), had the weakest correlations, though the correlation coefficients for all species were high (Table 1). Simple linear regression showed a significant relationship between inferred and absolute concentrations for each individual species (Fig. S4). The slope coefficient varied between species, with *L. iners* reporting the highest value (β_1_ = 0.88) and BVAB2 presenting the lowest (β_0_ = 0.59).

**TABLE 1:** Pearson correlation coefficients of single species between absolute concentration versus relative abundance and inferred concentration

Species	Pearson correlation coefficient	
	Relative abundance	Inferred abundance
*Megasphaera*	0.949	0.978
BVAB2	0.902	0.952
*Lactobacillus crispatus*	0.958	0.920
*Atopobium vaginae*	0.901	0.916
*Lactobacillus jensenii*	0.894	0.911
*Gardnerella vaginalis*	0.869	0.890
*Lactobacillus iners*	0.889	0.872

10.1128/mSystems.00777-19.4 FIG S4 Inferred concentration can predict absolute concentration. Scatter plot of inferred concentration versus absolute concentration. Both axes are plotted on a logarithmic scale. Inferred concentrations are significant predictors of absolute concentration with various intercept and slope coefficients between species. Title indicates parameters of the linear model *y* = β_0_ + β_1_. (a) L. crispatus; (b) L. jensenii; (c) *L. iners*; (d) *G. vaginalis*; (e) *Megasphaera*; (f) BVAB2; (g) *A. vaginae*. Download FIG S4, PDF file, 0.6 MB. Copyright © 2020 Tettamanti Boshier et al. 2020 Tettamanti Boshier et al. This content is distributed under the terms of the Creative Commons Attribution 4.0 International license.

**FIG 5:**
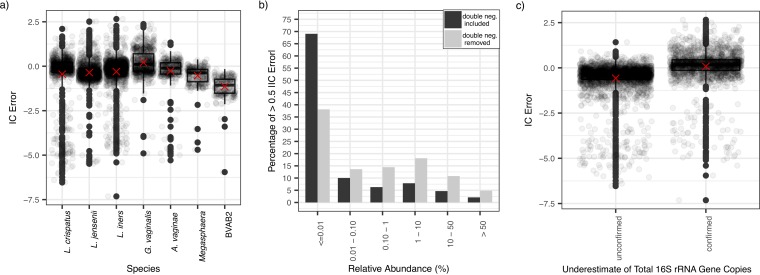
Low relative bacterial abundance is the major predictor of IC error for inferred concentrations compared to absolute concentrations. (a) Boxplots displaying IC error (equation 2), with zero inferred concentrations removed, indicate low IC error rates overall. Inferred values are consistent overestimates for BVAB2 and *Megasphaera* spp. Boxes indicate the interquartile range, whiskers are 1.5× the IQR, and dots are samples outside this range. Red crosses are means. (b) Bar chart of incidence of >0.5 IC error by relative abundance group. Black bars include double negatives (0 inferred concentration and threshold absolute concentration): 93% of >0.5 IC errors are accounted for by relative abundances of <10% (85% by relative abundances <1%). For the gray bars, concurrent negative samples are removed: 84% of >0.5 IC errors are accounted for by relative abundances of <10% (66% by relative abundances <1%). (c) Boxplots displaying the IC errors for samples with unconfirmed and confirmed underestimates of total bacterial load by broad-range qPCR assay (samples where BR16S is lower than the sum of concentrations of the seven targeted species). Data points with zero inferred concentrations were removed. Samples with underestimates of total bacterial load overestimate the single-species concentration more than do other samples. Overall, however, the range of IC error is comparable between both groups. Boxes are the interquartile range, whiskers are 1.5× the IQR, and dots are samples outside this range. Crosses indicate means.

We defined error of inferred concentration (IC) error as shown in equation 2. Although there was a large range in errors for nonzero inferred concentrations [Fig. 5a; range, −7.32 log_10_ (16S rRNA gene copies per swab) – 2.66 log_10_(16S rRNA gene copies per swab)], the mean IC error [−0.319 log_10_(16S rRNA gene copies per swab)] and standard deviations [0.999 log_10_(16S rRNA gene copies per swab)] were low. Moreover, the median IC error for most species approximated zero with samples within the interquartile range (IQR), demonstrating a minimal IC error (Fig. 5a). However, for BVAB2 and *Megasphaera*, the IQR of the IC error, while narrow, was all <0, implying consistent overestimation of the absolute concentration by the IC (pairwise *t* test, *P* < 0.05). There was also a trend toward global underestimation of *G. vaginalis* using inferred concentration (Fig. 5a).

### Low relative abundance is the major source of IC error.

The variance in the relationship with absolute concentration tended to be inversely proportional to species concentrations (Breusch-Pagan test; *P* = 0.06), highlighting that a larger range of IC errors tended to be reported at lower species-specific bacterial loads (Fig. 4b). Accordingly, 93% of >0.5 IC errors were accounted for by relative abundances below 10 and 85% by relative abundances below 1%. Many of these IC errors occurred on double negatives: samples for which the inferred concentration was zero and the absolute concentration was reported at threshold. When these samples were removed from the analysis, 84% of >0.5 IC errors were accounted for by relative abundances of <10, and 66% were accounted for by relative abundances below 1% (Fig. 5b). The median absolute concentration above the limit of detection for >0.5 IC errors was 5.95 log_10_(16S rRNA gene copies per swab) (IQR, 4.03 to 7.88; range, 1.97 to 10.39).

We defined false-positive samples as nonzero inferred concentration values when the absolute concentration qPCR values were at or below the detection threshold, and we defined false negatives as zero values for the inferred concentration when absolute concentrations were above the detection threshold. False negatives were more common (23.6% of samples) than false positives (3.17% of samples), which demonstrates that targeted qPCR is more sensitive for single species detection than is NGS.

The incidence of false negatives was not equal across species, with *G. vaginalis* having the highest percentage of false negatives, followed by *L. inners* and *A. vaginae* (L. crispatus, 13.8%; L. jensenii, 31.1%; *L. iners*, 35.1%; *G. vaginalis*, 60.4%; *A. vaginae*, 35.3%; *Megasphaera*, 5.40%; BVAB2, 9.84%). The higher percentages of false negatives for some species occurred because they are often present at moderate concentrations, near the relative abundance error threshold. The median qPCR value for false-negative samples was 3.92 log_10_(16S rRNA gene copies per swab) (IQR, 2.88 to 4.82; range, 1.97 to 7.84), again showing that IC errors generally occur at lower bacterial loads.

The total bacterial load measured by broad-range qPCR assay was frequently below the sum of the concentration of all seven species measured by targeted qPCR assays (37.6% per species per sample). Nonzero inferred single species concentrations from samples with underestimates of total bacterial load consistently overpredicted absolute concentration (one-tailed *t* test, *P* < 2.6e–4) and did so more than at other points (pair-wise *t* test, *P* < 2.2e–16) (Fig. 5c). Nonzero inferred concentrations from samples with suspected underestimates of total bacterial load (samples where BR16S is lower than the sum of concentrations of the seven targeted species) had a median IC error of 0.171 log_10_(16S rRNA gene copies per swab) (IQR, –0.138 to 0.447; range, –7.31 to 2.66) compared to −0.368 log_10_(16S rRNA gene copies per swab) (IQR, −0.638 to −0.143; range, −6.54 to 1.42) in other samples.

L. crispatus had the highest percentage of false positives (L. crispatus, 8.42%; L. jensenii 1.08%; *L. iners*, 3.56%; *G. vaginalis*, 0.46%; *A. vaginae*, 3.07%; *Megasphaera*, 1.12%; BVAB2, 1.79%). The median relative abundance of false positives across all samples was extremely low at 0.06% (IQR, 0.04 to 0.11%; range, 0.0007 to 36.8%).

### Concentrations inferred from NGS predict observed absolute concentration regardless of sample diversity or sequencing depth.

**FIG 6:**
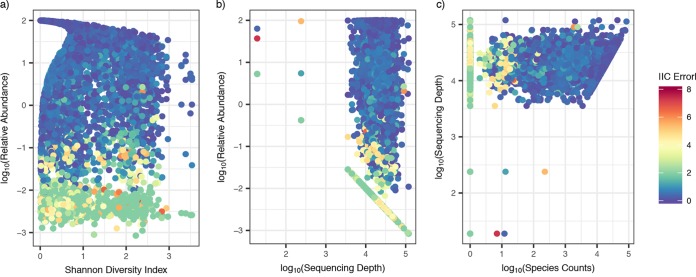
Sample diversity, sequencing depth, and species counts do not impact the IC error of the inferred concentration. Scatterplots color-coded by IC error are shown. Each dot is a sample for a specific species from a single participant. (a) Relative abundance versus Shannon diversity index. A high IC error predominantly occurred at a low relative abundance but across both low and high diversity samples. (b) Relative abundance versus sequencing depth. A high IC error predominantly occurred at a low relative abundance but across various levels of sequencing depth. (c) Sequencing depth versus species counts. A high IC error occurred at species counts below 100, although a >0.5 IC error is observed across all species counts.

Inferred concentrations did not disproportionally record misleading results from low- or high-diversity samples, as measured by the Shannon diversity index (Fig. 6a). Moreover, we observed occasional large absolute IC errors across all sequencing depths (Fig. 6b). Low bacterial abundance was the primary source of absolute IC error regardless of diversity or sequencing depth (Fig. 6a and b). A >0.5 absolute IC error was observed across all raw species counts, but the largest absolute IC errors (>2) were almost exclusively associated with raw species counts below 100 (Fig. 6c).

### Inferred concentration estimates are predictive of most temporal changes in single species bacterial load.

**FIG 7:**
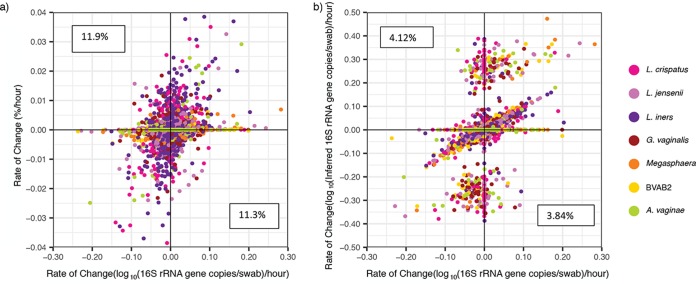
Inferred concentrations allow somewhat accurate inference of kinetic changes between two sequential samples. (a) Scatterplot of the change in the absolute abundance versus the change in the relative concentration shows poor correlation. Pearson correlation coefficient (PCC): *r* is 0.271 and *P* < 2.2e–16. A high percentage of the observed changes in the relative abundance is in the opposite direction to those in the absolute concentration (left-upper and right-lower error quadrants marked with percentages). (b) A scatterplot of the absolute concentration versus the inferred concentration shows improved correlation. Both axes are plotted on a logarithmic scale. PCC: *r* is 0.392 and *P* < 2.2e–16. The percentages correspond to the number of data points that fall within the error quadrants and are lower than for the relative abundance. The inferred values misreport the direction of kinetics less frequently.

We next examined whether inferred concentration is a useful tool for evaluating individual species kinetics by determining changes in bacterial levels over the course of a day. The rates of change in relative abundances correlated only weakly with absolute concentrations (*r* = 0.271, *P* < 2.2e–16). Moreover, 23.2% of the time, we observed a change in relative abundance in the opposite direction to that of absolute concentration (see the top-left and bottom-right quadrants of Fig. 7a). This type of error occurred commonly for both the most abundant (e.g., L. crispatus) and rarer species (e.g., BVAB2).

The rates of change in the inferred concentration showed improved correlation with the rates of change in the absolute concentration (*r* = 0.392, *P* < 2.2e–16). The mean rIC error (defined in Materials and Methods) was low [−2.71 × 10^−3^, standard deviation (SD) = 1.54 log_10_(16S rRNA gene copies per swab) per hour], though the range of rIC errors was high [−9.29 to 9.31 log_10_(16S rRNA gene copies per swab) per hour], indicating occasional samples with very poor prediction. The inferred concentrations decreased the sign rIC error rate by >50% (from 23.2 to 7.97%, Fig. 7b).

**FIG 8:**
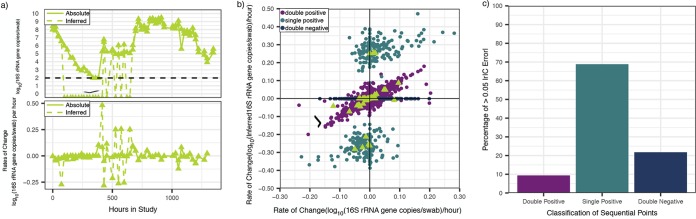
Inferred concentration measures allow accurate inference of kinetic changes between two sequential nonnegative samples. (a, top) Levels of *A. vaginae* over time in a single participant (dotted is inferred and solid is absolute concentration); (bottom) rate of change in levels of *A. vaginae* over time in the same participant (dotted is inferred and solid absolute concentration). The divergence in swab-to-swab levels between inferred and absolute concentrations varies only when the inferred concentration is zero in one of the sequential samples. (b) Scatterplot of rate of change of inferred concentration as predicted by NGS versus qPCR observed values. Both axes are plotted on a logarithmic scale. The data are the same as in Fig. 7a and b. The triangles correspond to panel a. Points are colored according to whether consecutive samples were double positive (both >0 inferred concentration), single positive (one >0 and one 0 inferred concentration), or double negative (both 0 inferred concentration). Data points in which both samples are positive (no zeroes) are much more highly correlated (*r* is 0.876 and *P* < 2.2e–16). (c) A majority of rIC errors > 0.05 occur during transitions between positive and negative samples (single positives).

Figure 8a shows a typical profile of *A. vaginae* absolute levels and sample-to-sample change to demonstrate the two types of rIC errors that were most common to the data. The first were large positive or negative rates which occurred when one of two consecutive points had an inferred concentration of zero (single positives), while the absolute concentration was detectable by qPCR. These points resulted in dramatic overestimation of growth or contraction rates for individual species across all samples (Fig. 7b and 8b, right-upper and left-lower quadrants). Such rIC errors often occurred when species were transitioning to or from a low concentration (<10^6^ 16S rRNA gene copies per swab). The second type of rIC error occurred when two consecutive points had inferred concentrations of zero (double negatives), resulting in underestimation of growth or contraction rates for individual species (Fig. 8b). This phenomenon also commonly occurred when a species was transitioning to or from a low concentration (<10^6^ 16S rRNA gene copies per swab). These two forms of transitions accounted for 91.7% of rIC error > 0.05 (Fig. 8c). If all transitions involving a zero value were eliminated from the analysis, we observed excellent correlation between inferred and observed rate of change (*r* = 0.876, *P* < 2.2e–16; Fig. 8b). It follows that inferred concentrations do not capture kinetics during microbial blooming or contraction, when bacteria are at low concentration or not detected using the less sensitive broad-range PCR with NGS approach. However, inferred concentrations can be used to estimate individual species growth and contraction rates when bacteria are present at higher concentrations, such as >10^6^ 16S rRNA gene copies per swab.

### Complete linkage clustering by inferred and absolute concentrations shows general agreement.

To assess whether inferred concentrations provide similar or disparate classification of samples, we clustered samples using complete linkage hierarchical clustering based on Euclidean distances (19) by inferred and absolute concentrations of the seven species for which the two measures are available (Fig. S5). We compared the resulting dendrograms using the entanglement coefficient from the dendextend package in R (20), where a value of 1 corresponds to complete discordance and a value of 0 indicates perfect alignment. The two dendrograms were found to be in agreement, with a low entanglement coefficient 0.11.

10.1128/mSystems.00777-19.5 FIG S5 Complete hierarchical linkage clustering by 7 key species across all samples. (a) Absolute concentration; (b) inferred concentration. The two analyses are somewhat coherent with one another. Although the two dendrograms are in agreement with a low entanglement coefficient (0.11), the measurements identify a different number of clusters. The third cluster identified by inferred concentration appears to be primarily distinguished from other samples dominated by *Lactobacillus* species (cluster 1) by having a low concentration of L. crispatus relative to other samples dominated by *Lactobacillus* species. This occurs as the inferred concentrations can report lower concentrations than the threshold of detection reported by absolute concentrations. Download FIG S5, PDF file, 0.7 MB. Copyright © 2020 Tettamanti Boshier et al. 2020 Tettamanti Boshier et al. This content is distributed under the terms of the Creative Commons Attribution 4.0 International license.

We next determined the number of clusters using NbClust package in R (21). The absolute concentration identifies two, whereas the inferred concentration identifies three clusters. The third cluster arose from a general distinction between samples dominated by L. crispatus from *L. iners* as the inferred concentrations had a lower threshold (1 16S rRNA gene copy per swab) than the qPCR (93.8 16S rRNA gene copies per swab).

### Inferred concentration may provide the most comprehensive overview of individual species kinetics.

Inferred concentrations can be calculated for all species captured by NGS. In Fig. 1 and Fig. S1, we show the inferred concentrations of the most abundant species across all samples. We imposed a 1% relative abundance threshold to limit the possible 0.5 IC error described in Fig. 5b. This relative abundance cutoff results in abrupt appearance and disappearance of organisms. Although we cannot validate our projections for species outside the seven key bacterial species for which we have targeted qPCR assays, inferred concentrations have the potential to describe the kinetics of relevant species present at moderate to high concentrations during bacterial shifts in the microbiome.

We carried out complete linkage hierarchical clustering based on the Euclidean distance by inferred concentration and relative abundance for the 20 most abundant species of the data set (Fig. S6). The resulting dendrograms showed general agreement, with an entanglement coefficient of 0.12. Both techniques identified two clusters defined by high-concentration *G. vaginalis* and high diversity versus *Lactobacillus* predominance (21).

10.1128/mSystems.00777-19.6 FIG S6 Complete hierarchical linkage clustering using the top 20 most abundant species across all samples. (a) Relative abundance; (b) inferred concentration. The two analyses are coherent with one another. Specifically, the two dendrograms are in agreement with a low entanglement coefficient (0.12), and both measurements identify the same number of clusters. Download FIG S6, PDF file, 1.3 MB. Copyright © 2020 Tettamanti Boshier et al. 2020 Tettamanti Boshier et al. This content is distributed under the terms of the Creative Commons Attribution 4.0 International license.

## DISCUSSION

An ideal assay that characterizes bacterial communities in an ecological niche would capture several metrics, including species composition, diversity, and quantity, as reflected by the absolute concentrations of all species present. Broad-range PCR of phylogenetically informative genes, followed by NGS, is the most commonly used approach and captures the first two metrics. However, because total bacterial levels may shift dramatically over narrow time intervals, relative abundance measures by NGS do not reflect absolute concentration. Although it is possible to circumvent this issue with targeted (taxon-specific) qPCR, these assays are expensive, time-consuming, and only available in specialized laboratories. Invariably, the absolute concentration of many relevant species is left unmeasured due to these constraints.

This measurement gap is highly relevant to clinical studies of the human microbiome, in which the total bacterial load may not be stable. It is biologically plausible that the absolute levels of critical species are more predictive of health and disease states than relative levels, as is the case with classical single-pathogen infectious diseases. Moreover, serial measurements of absolute levels are necessary to fully capture nonlinear microbial dynamic changes which relate to interspecies competition for limited resources.

Using a large longitudinal data set of the vaginal microbiome notable for frequent changes between low and high diversity states, we demonstrate that the absolute concentration of a given species can be inferred by multiplying the total bacterial quantity by its relative abundance as measured by NGS. Given that quantitating total bacterial load is affordable and available to many laboratories, this simple approach may allow estimation of absolute concentration without needing to perform qPCR on all samples.

Our technique is remarkably predictive of absolute concentration with certain key exceptions. Species such as BVAB2 and *Megasphaera*, which were often present at low absolute concentrations, were notable for high precision but slight inaccuracy: inferred concentration consistently slightly overestimated the abundance for these species. This result highlights that individual comparisons between inferred and absolute concentration must be considered for all species of interest. Other than in an exploratory fashion, we do not advocate the use of inferred concentration for species that have not been validated in depth with targeted qPCR assays and compared to the absolute concentration.

Second, our approach has a very high IC error rate when the relative abundance is low or zero. In our qPCR data set, low-level colonization of certain species often precedes a surge in levels prior to this species predominating. Because qPCR is more sensitive than NGS for small amounts of bacterial DNA and because inferred concentration relies on NGS, the inferred concentration will often miss persistent low-level colonization, as well as the critical early growth phase or late contraction phase of relevant species. Despite this fact, the inferred concentration performs remarkably well at estimating growth and decay rates at the single species level, provided these rates are estimated based on positive sequential samples. One might be able to improve the accuracy of the inferred concentrations by increasing the sequencing depth or improving the accuracy of measurements of the total bacterial load.

A final issue not addressed by our technique is the limitation inherent to comparing bacterial quantities between species using qPCR based on differing amplification efficiencies of different assays. This variability may arise from different bacterial targets having various GC contents, secondary structures, and amplification product sizes. In this sense, absolute concentration by qPCR may not be a perfect gold standard for comparing inferred concentration.

Further work is needed to validate the use of inferred concentrations, including the identification of the relative abundance threshold above which it is accurate, in other microbiota samples. In our study, the primers used to measure total bacterial loads target the same region, V3-V4, that we use for the NGS assay. The concordance that we see may not be applicable when there are differences in the variable regions used for measuring total bacterial concentrations and for NGS.

In summary, we developed and validated a simple, user-friendly method to estimate absolute species concentration in complex polymicrobial vaginal communities. This method is best employed when species are present at a >10% relative abundance and must be validated for each species of interest. Ultimately, the inferred concentration of one or several species may serve as a more predictive variable of disease association, compared to relative abundance, and may advance our understanding of how specific environmental and host factors influence microbial concentrations.

## MATERIALS AND METHODS

### Ethics statement.

Vaginal samples were collected using protocol 417, which was approved by the institutional review board (IRB) at the University of Washington (approval STUDY00000398). All participants provided written informed consent prior to study enrollment. The study was approved by the IRB as part of protocol 417.

### Study population.

The study population was comprised of 20 women enrolled in a longitudinal study of bacterial vaginosis (BV) natural history at the University of Washington Virology Research clinic between 2015 and 2017. At enrollment, participants were given sufficient swabs for three times daily swabs over 60 days for self-collection of vaginal swabs. Diagnosis, sample collection, storage, and processing of swabs are as described in (22). Participants were also given a study diary to record vaginal odor and discharge, two symptoms that are characteristic of BV, antibiotic use, menstruation, sexual activity, and other medical events. Participants returned a median of 160 vaginal swabs, and we analyzed 1,320 data points for each of the seven key species.

### DNA extraction and quantitative PCR.

DNA was extracted from vaginal swabs using the BiOstic Bacteremia DNA isolation kit (Mobio, Carlsbad, CA). Sham swabs without human contact were extracted in parallel to assess contamination from reaction buffers or the collection swabs. No template water controls were included to determine whether there was any contamination from PCR reagents. Each sample was evaluated for PCR inhibition (23), and total bacterial concentrations in each sample were measured using a qPCR assay that targets the V3-V4 region of the 16S rRNA gene of most bacteria, including the seven bacterial species evaluated in this study (24). Concentrations of specific vaginal bacteria were measured using qPCR assays targeting seven key vaginal bacteria: Atopobium vaginae, BV-associated bacterium 2 (BVAB2), Gardnerella vaginalis, Lactobacillus crispatus, Lactobacillus jensenii, Lactobacillus iners, and *Megasphaera* (combined species 1 and 2), species originally developed in other studies (12, 24, 25). The primers, probes, and assay conditions are listed in Table S1 in the supplemental material. For qPCR assays using standard cycling, each 15-μl reaction mixture contained 1× buffer A (Life Technologies, Carlsbad, CA), 3 mM magnesium chloride, a 1 mM deoxynucleoside triphosphate blend containing dUTPs, 0.8 μM concentrations of each primer, 150 to 300 nM probe, 0.03 U of uracil-*N*-glycosylase, and 0.3 to 1.0 U of AmpliTaq Gold DNA polymerase (Life Technologies). For qPCR assays using FAST cycling, each 15-μl reaction mixture contained 1× TaqMan Fast Advanced Master Mix (Life Technologies), 0.8 μM concentrations of each primer, and 150 to 200 nM probe. Assays were run on a QuantStudio 6 instrument (Life Technologies) in a 384-well format. The limit of quantification for the seven specific vaginal bacterial assays is 2.5 16S rRNA gene copies per swab with a linear range to 10^8^ 16S rRNA gene copies per swab. The BR-16S rRNA gene qPCR has a limit of quantification of 10 16S rRNA gene copies per swab with a linear range to 10^8^ 16S rRNA gene copies per swab as described previously (12, 24, 25).

10.1128/mSystems.00777-19.7 TABLE S1 Primers, probes, and assay conditions. Download Table S1, XLSX file, 0.01 MB. Copyright © 2020 Tettamanti Boshier et al. 2020 Tettamanti Boshier et al. This content is distributed under the terms of the Creative Commons Attribution 4.0 International license.

We measured the relative abundances of bacterial taxa using broad-range PCR targeting the V3-V4 region of the 16S rRNA gene with NGS on the Illumina MiSeq instrument (Illumina, San Diego, CA) (26). The *DADA2* pipeline was used to infer sequence variants from raw reads for subsequent analysis (27). Sequences were classified using the phylogenetic placement tool pplacer (28) and a curated reference set of vaginal bacteria (8). The median sequencing depth was 23,304 reads (IQR, 16,237 to 31,292.5; range, 19 to 118,436). Only two samples had sequencing depths below 1,000; the remaining samples were all above 3,540. We used NGS to refer to data generated using broad-range PCR and sequencing. The sequence reads have been submitted to the NCBI Short Read Archive (SRA; BioProject PRJNA549339). The relative abundances and absolute concentrations of specific vaginal bacteria were measured on all samples in two participants and in daily morning samples for the remaining 18. We performed qPCR on all samples collected from each participant, but for the purpose of this work we only consider the morning samples.

All data generated or analyzed during this study are included in the supplemental material (Tables S2, S3, and S4).

10.1128/mSystems.00777-19.8 TABLE S2 qPCR measurements of seven species*:* L. crispatus, *L. iners*, L. jensenii, *G. vaginalis*, *A. vaginae*, BVAB2, and *Megasphaera* and total bacterial load (BR16S). Download Table S2, CSV file, 0.1 MB. Copyright © 2020 Tettamanti Boshier et al. 2020 Tettamanti Boshier et al. This content is distributed under the terms of the Creative Commons Attribution 4.0 International license.

10.1128/mSystems.00777-19.9 TABLE S3 Relative abundance measurements of seven species: L. crispatus, *L. iners*, L. jensenii, *G. vaginalis*, *A. vaginae*, BVAB2, and *Megasphaera*. Download Table S3, CSV file, 0.1 MB. Copyright © 2020 Tettamanti Boshier et al. 2020 Tettamanti Boshier et al. This content is distributed under the terms of the Creative Commons Attribution 4.0 International license.

10.1128/mSystems.00777-19.10 TABLE S4 Taxonomic assignments and raw counts of all samples. Download Table S4, CSV file, 1 MB. Copyright © 2020 Tettamanti Boshier et al. 2020 Tettamanti Boshier et al. This content is distributed under the terms of the Creative Commons Attribution 4.0 International license.

### Statistical considerations.

We calculated inferred concentrations using equation 1:

(1) $$\begin{array}{l} \text{IC (16S rRNA gene copies/swab) = }\\ \text{RA (\%) }\times \text{ TBL (16S rRNA gene copies/swab)} \end{array}$$

where IC is the inferred concentration, RA is the relative abundance, and TBL is the total bacterial load. We present many of the plots and related calculations on a log_10_ scale. To keep all values finite when working with a log_10_ scale, the zero relative abundance (%) was mapped to 1/(sequencing depth). Zero inferred concentrations were mapped to 1. The choice of this mapping can impact some of the numerical results presented here, namely, the correlation coefficient and the clustering class of the samples. However, the general observations are consistent with any sensible choice of mapping.

We employed the smooth.fft function (19) to impose a low-pass filter to isolate the variance in our longitudinal qPCR data sets. The technique uses Fourier transforms to recognize and remove high-frequency signals. We assumed the high frequencies to be noise generated by either sampling or laboratory variability. For the results contained here, we apply a 25% filter, although we have found the results to be consistent across several different thresholds.

We defined the error of inferred concentration (IC error) according to equation 2 as follows:

(2) $${\text{IC error = log}}_{\text{10}}\text{(AC) }-{\text{ log}}_{\text{10}}\text{(IC)}$$

where IC is the inferred concentration, and AC is the absolute concentration. The rates of change per day were calculated between any two consecutive time points that were 18 to 36 h apart. Rates were calculated from log_10_ converted values for relative abundance and inferred and absolute concentration. We defined the error in rates from inferred concentrations (rIC error) as follows:

(3) rIC error = rates(AC) − rates(IC)

where IC is the inferred concentration, and AC is the absolute concentration. Comparison of the means was done using the t.test function in R (19). We used Pearson’s correlation coefficient and linear regression for all correlation analysis. This was done using the lm.test and cor.test function in the stats package in R (19). We denote the gradient and intercept of this model as β_0_ and the gradient as β_1_. Pearson’s correlation coefficients were compared using the Cocor package in R (18). The suite provides 10 tests for overlapping correlations, i.e., measurements taken from the same data set. All tests were significant, but we report the value of the Hittner test here for simplicity.

The Breusch-Pagan test was used to test the heteroskedasticity of the linear regression model of the relative abundance and inferred concentration versus the absolute concentration. It tests whether the variance of the errors from a regression is dependent on the values of the independent variables. This was implemented using the bptest of the lmtest package in R (29).

We constructed the dendrograms for clustering analysis by complete linkage hierarchical clustering of species abundance and/or concentration based on Euclidean distance between all sample pairs. We tested concordance between pairs of dendrograms using the entanglement coefficient found in the dendextend package in R (20). To calculate the coefficient, all of the samples are first numbered in the order they appear for each tree. The coefficient is then calculated by taking the Euclidean distance of these two vectors, which is then normalized by the worst-case entanglement value (i.e., the Euclidean distance when the order of the two dendrograms is opposite). The entanglement coefficient thus defined ranges from 0 to 1, with “0” indicating perfect alignment between the dendrograms and “1” indicating a complete mismatch.

### Data availability.

The relative abundance and absolute concentration for the seven species compared here can be found in Tables S2 and S3, respectively. The raw counts from high-throughput sequencing can be found in Table S4. Sequence reads are available on the NCBI Short Read Archive (BioProject PRJNA549339).
