# Supplementary material for: Complementing 16S rRNA Gene Amplicon Sequencing with Total Bacterial Load To Infer Absolute Species Concentrations in the Vaginal Microbiome
Source: mSystems. 2020 Apr 7;5(2):e00777-19. doi: 10.1128/mSystems.00777-19 (PMC7141891; doi:10.1128/mSystems.00777-19)

Participant 01

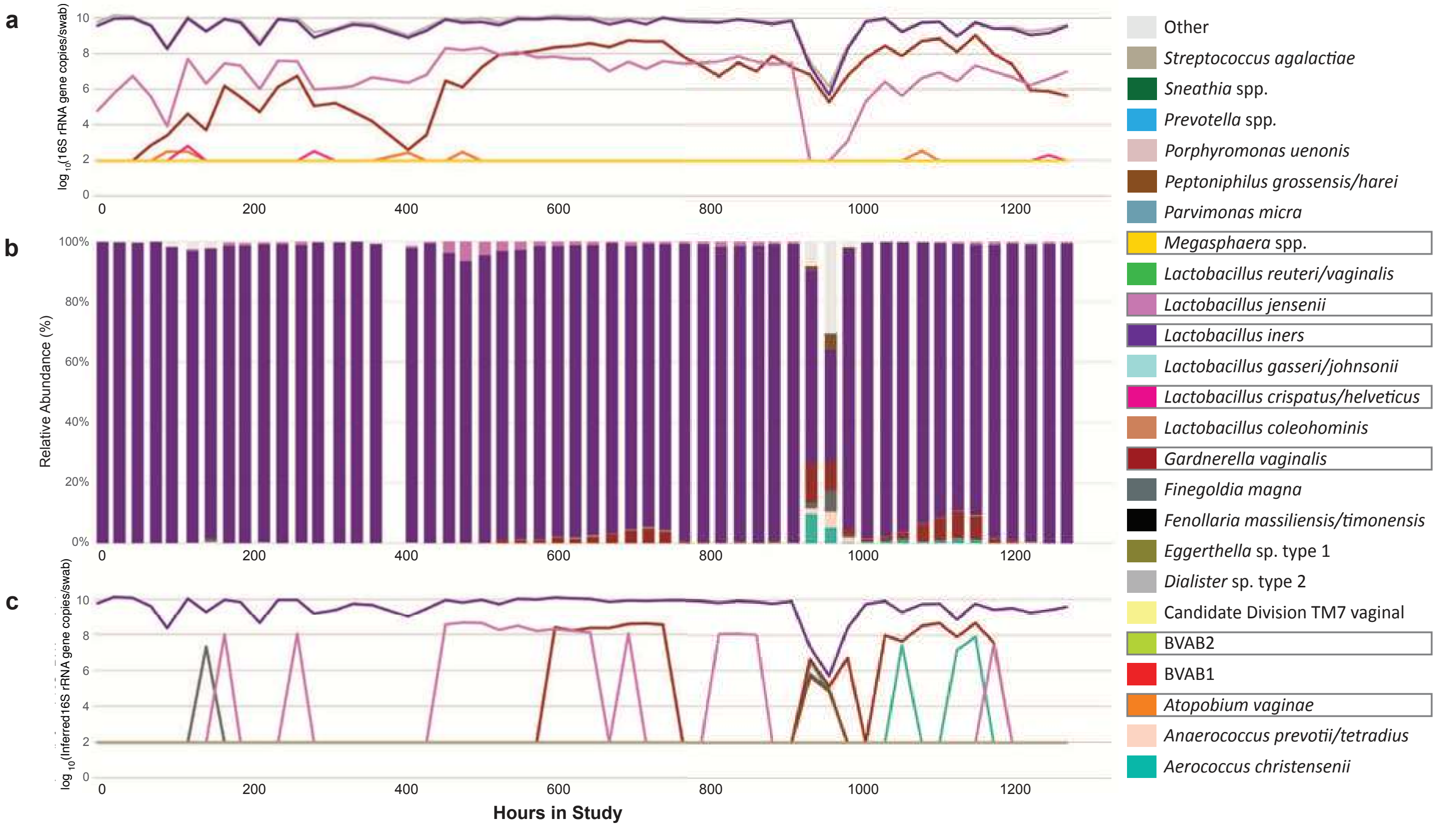

Participant 02

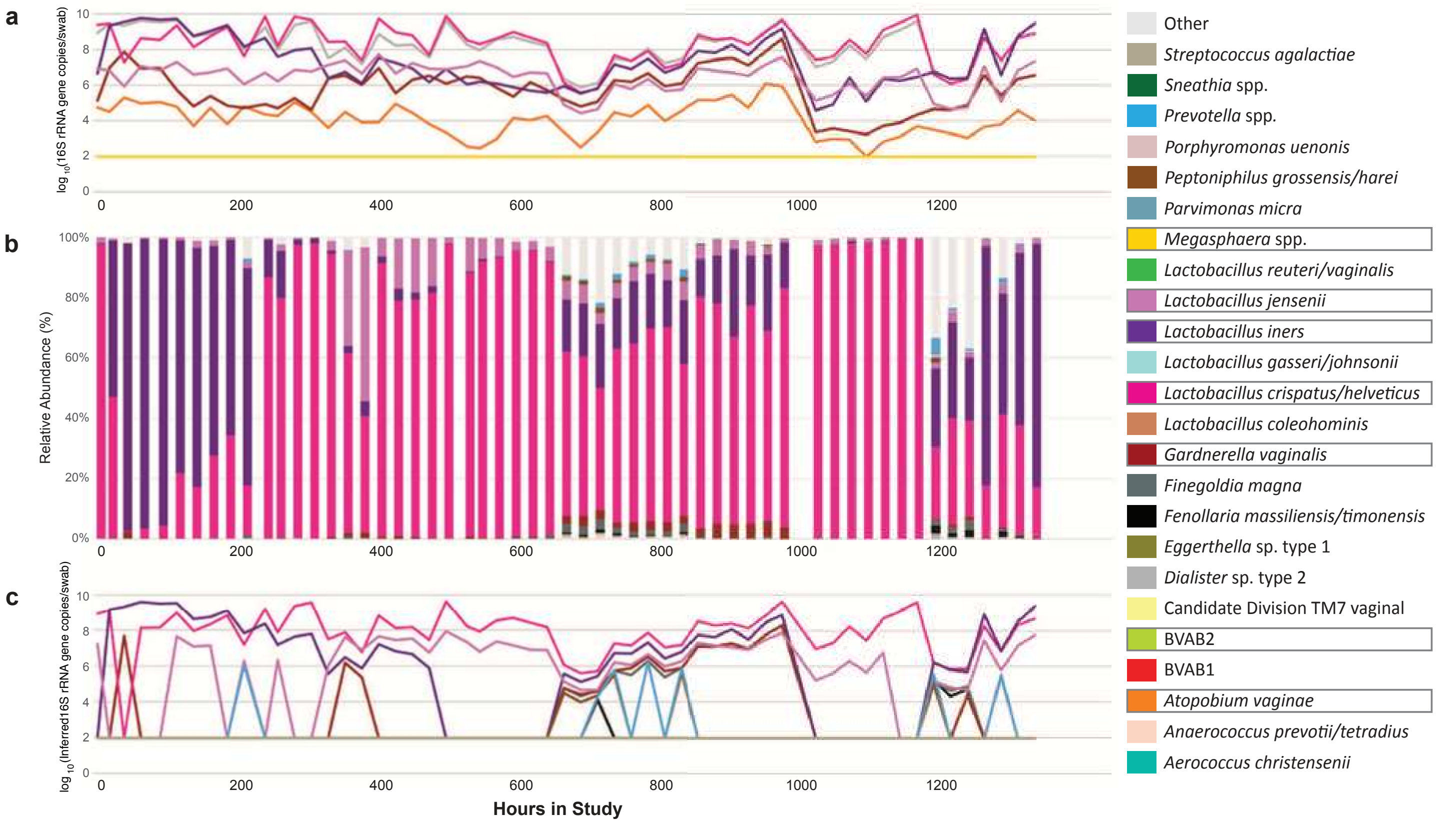

Participant 03

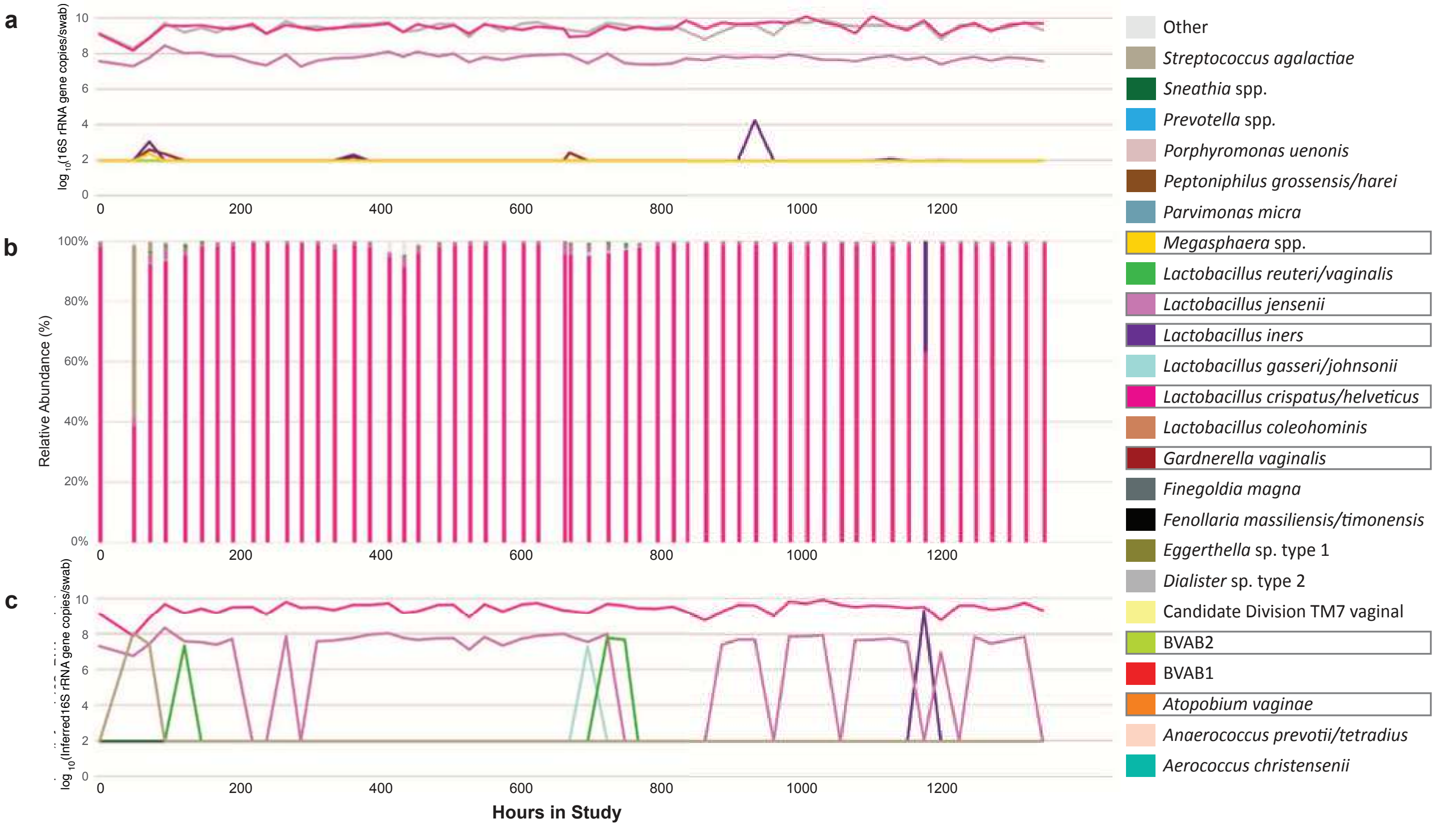

Participant 04

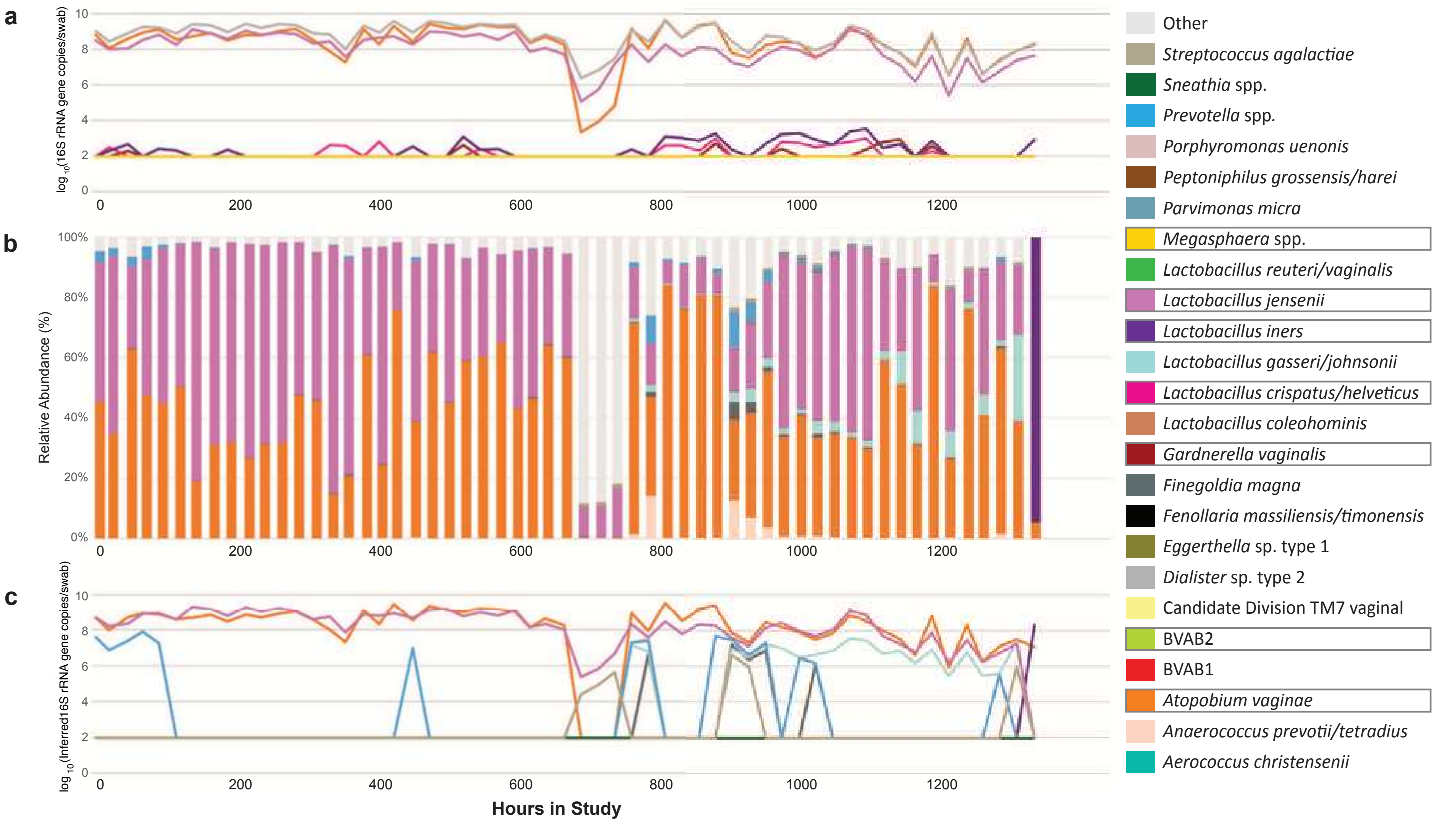

Participant 05

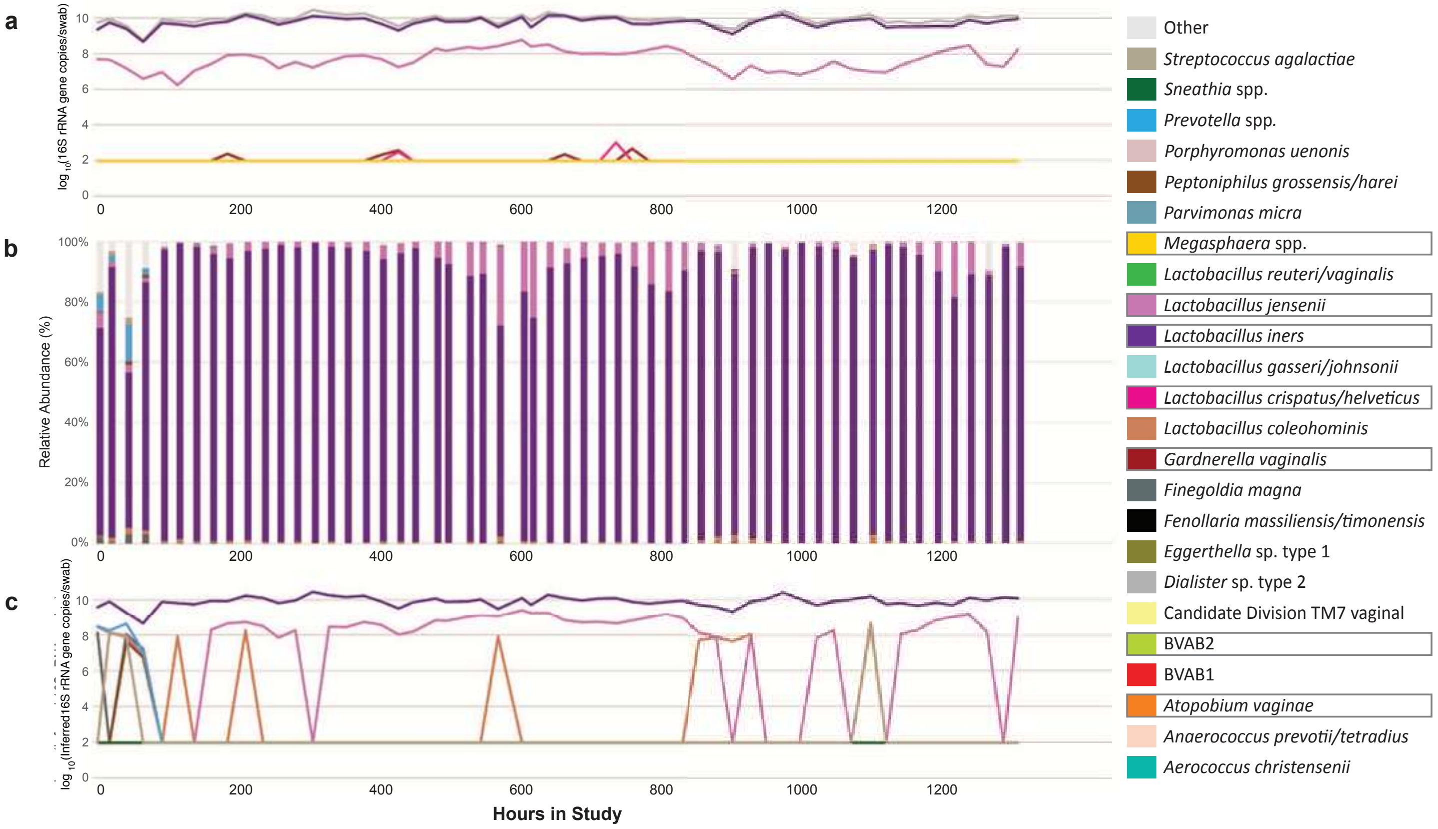

Participant 06

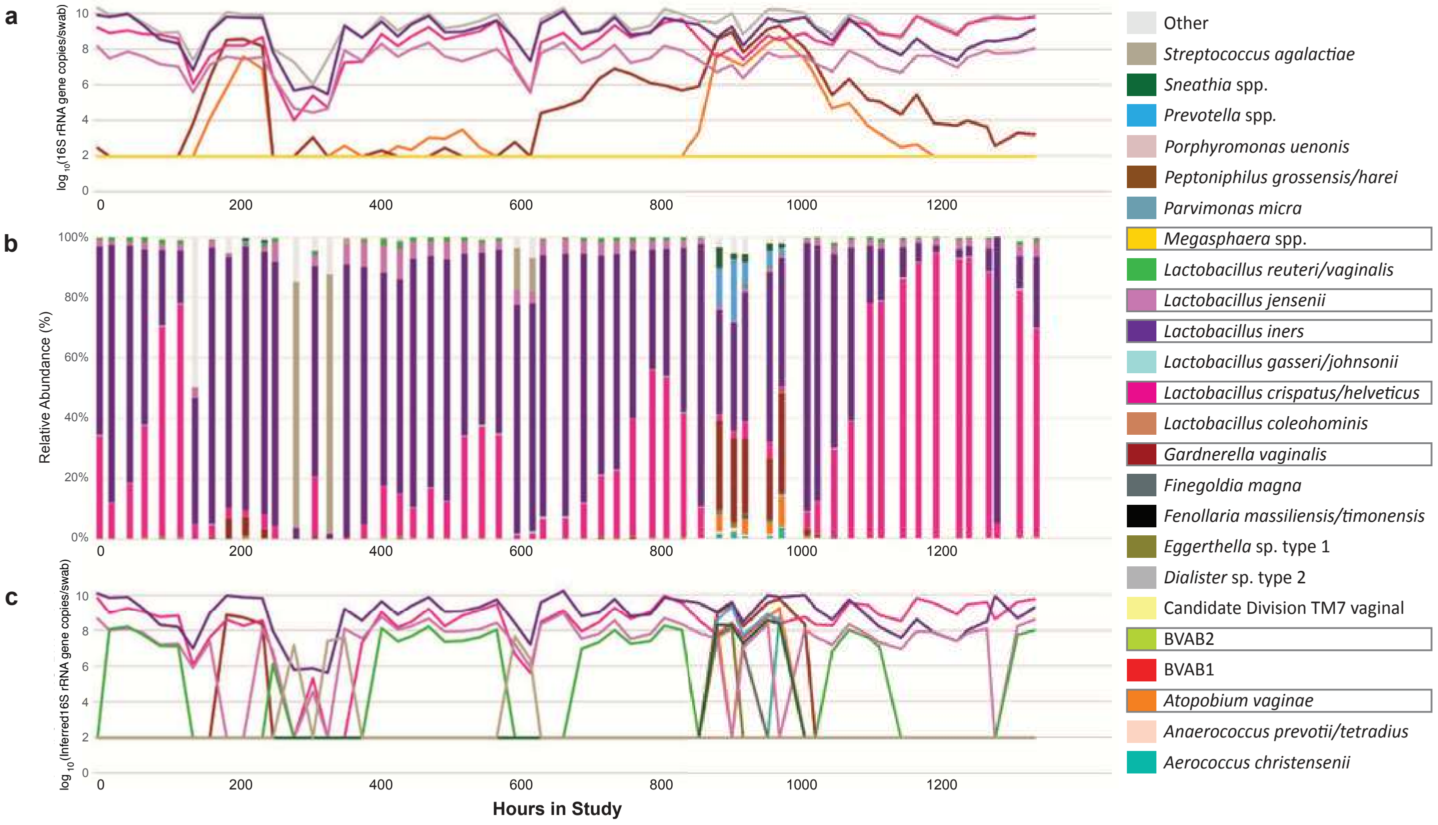

Participant 07

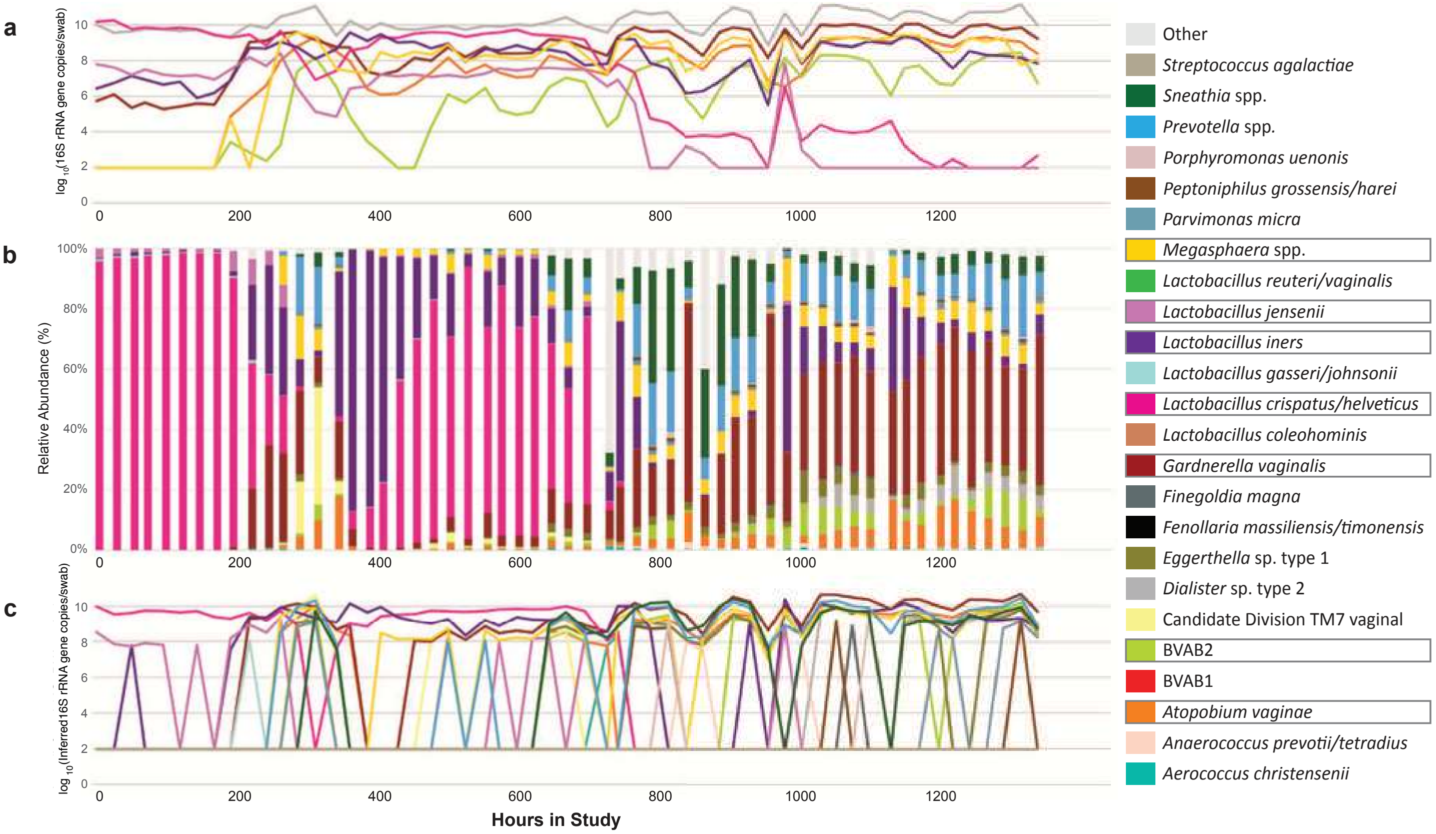

Participant 08

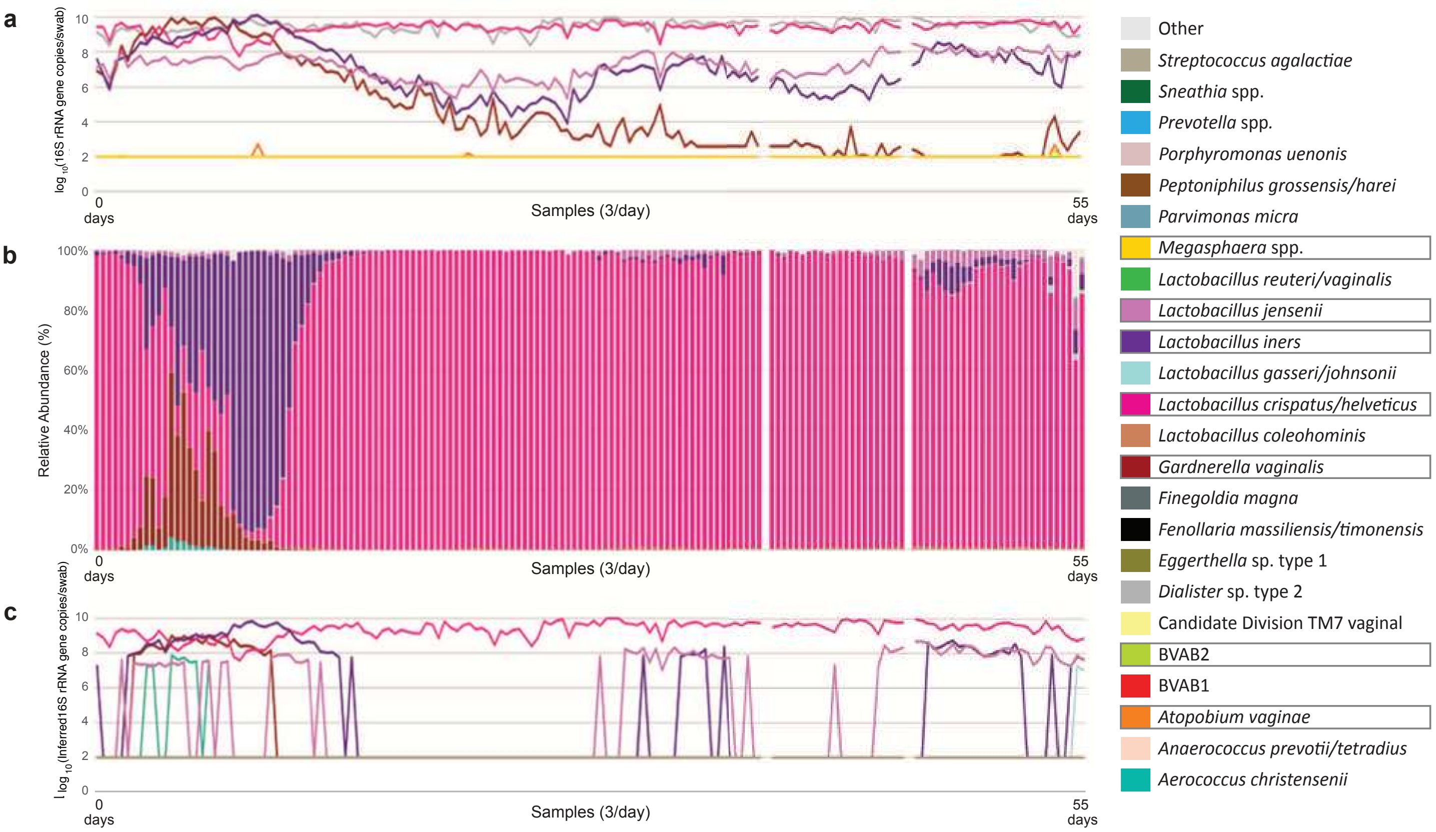

Participant 09

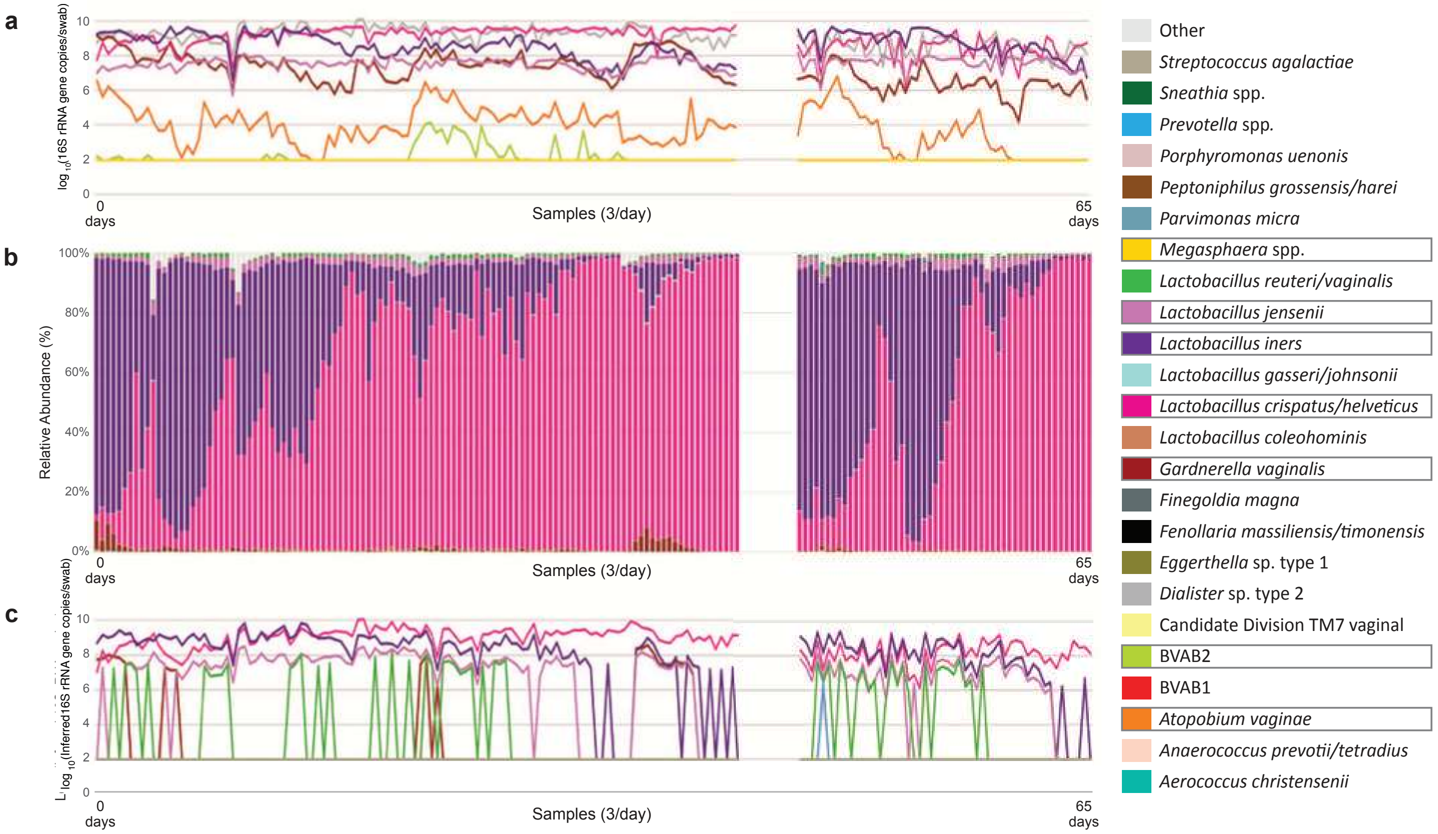

Participant 10

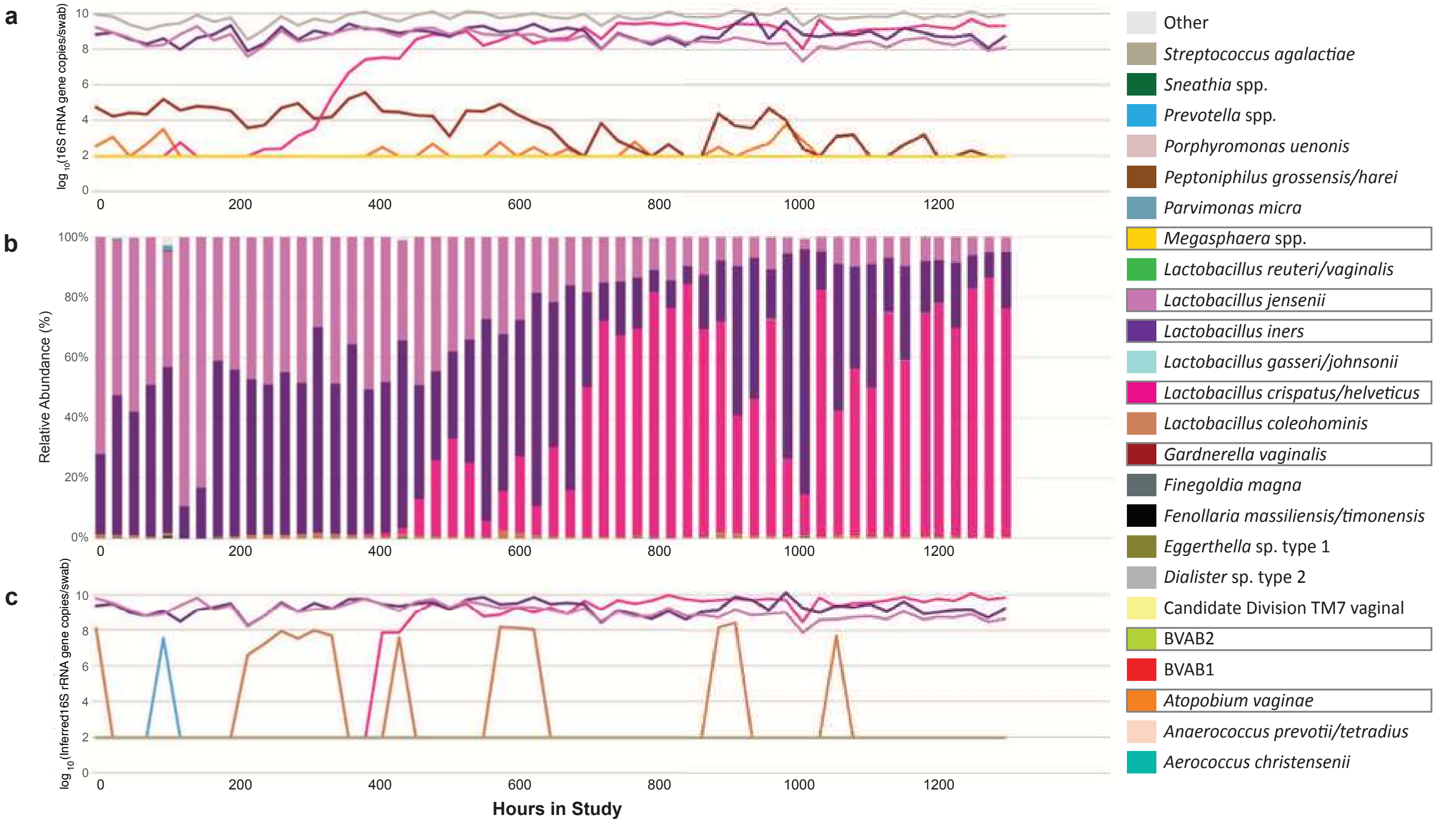

Participant 11

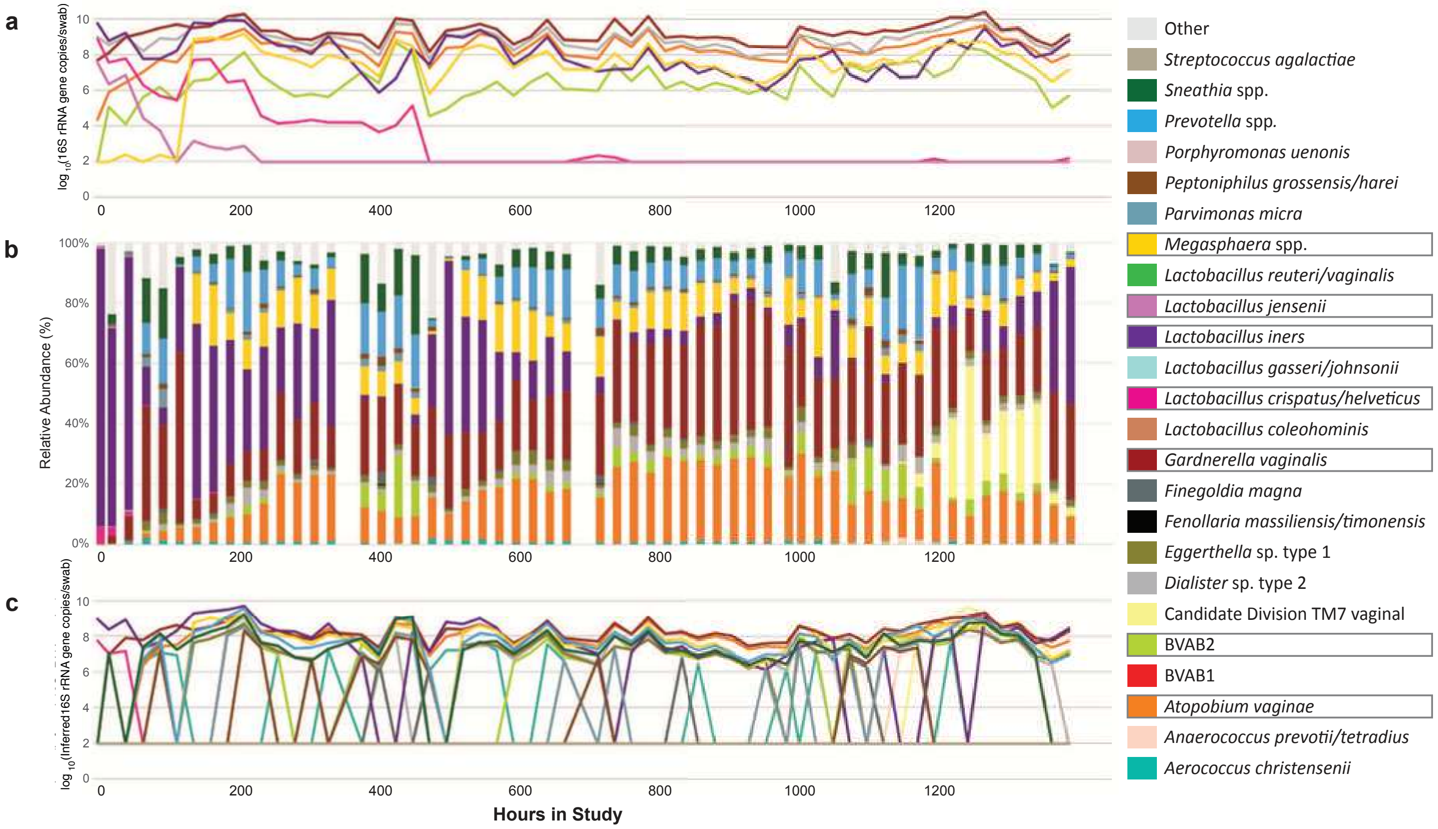

Participant 12

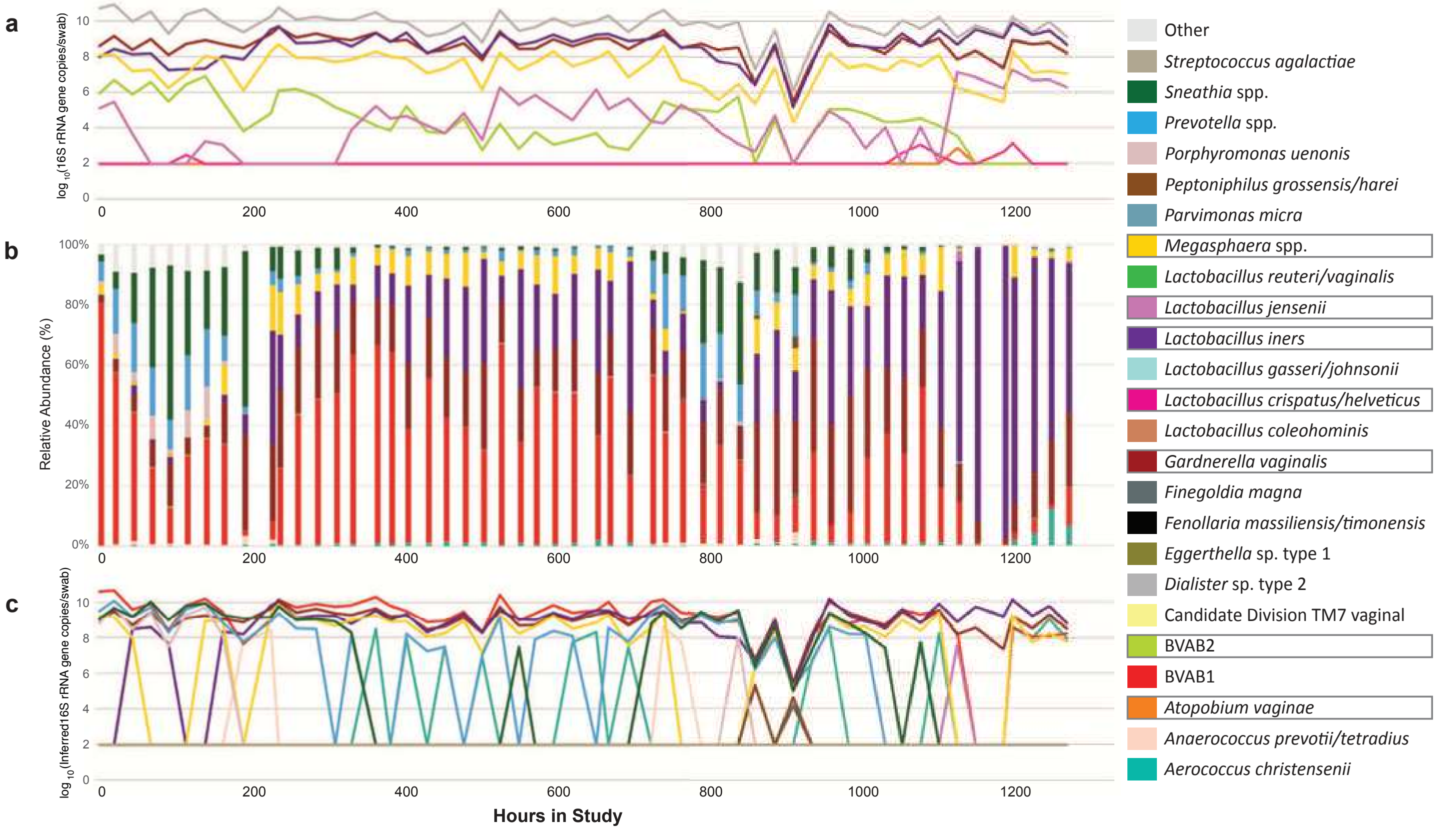

Participant 13

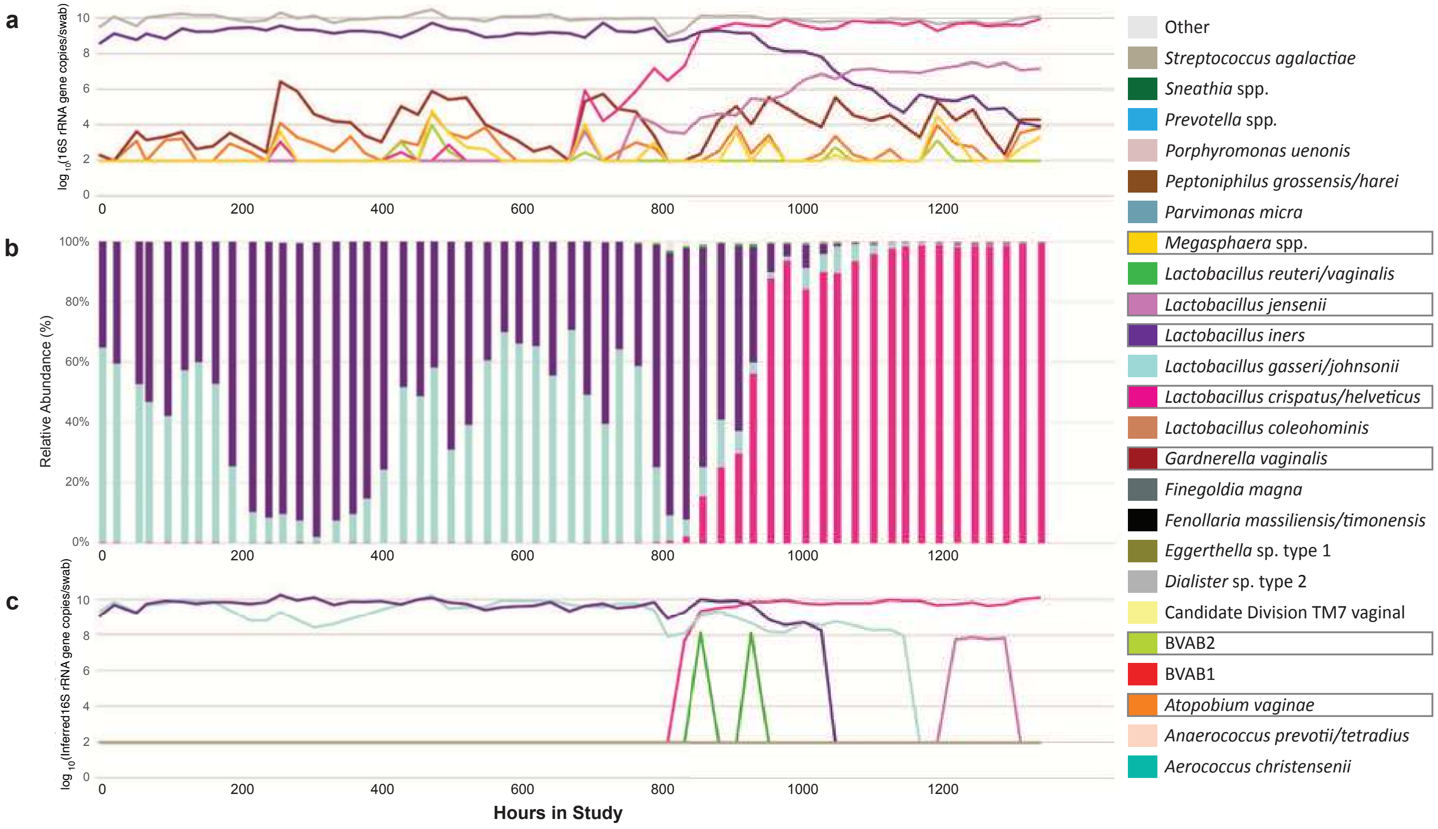

Participant 14

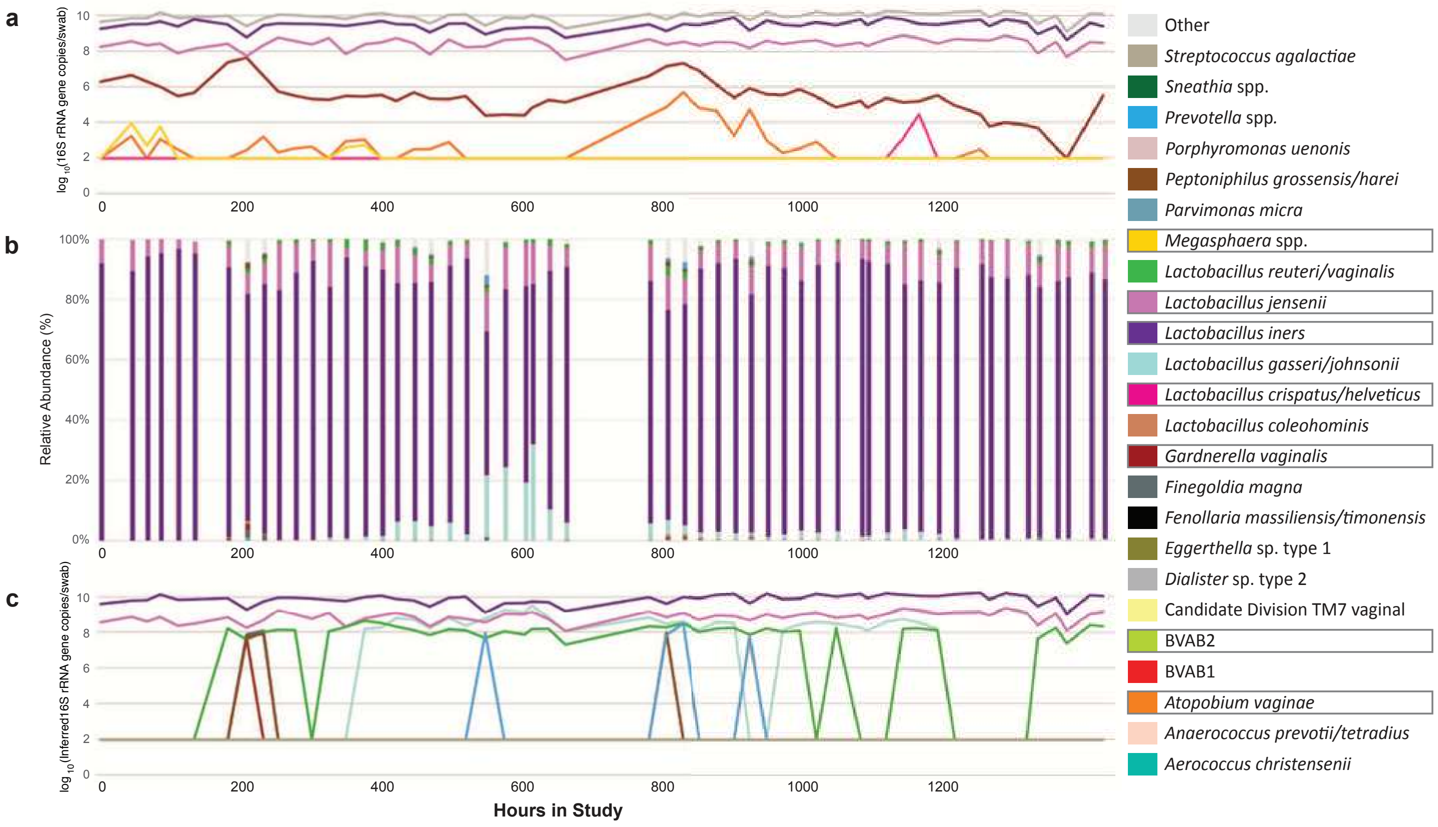

Participant 15

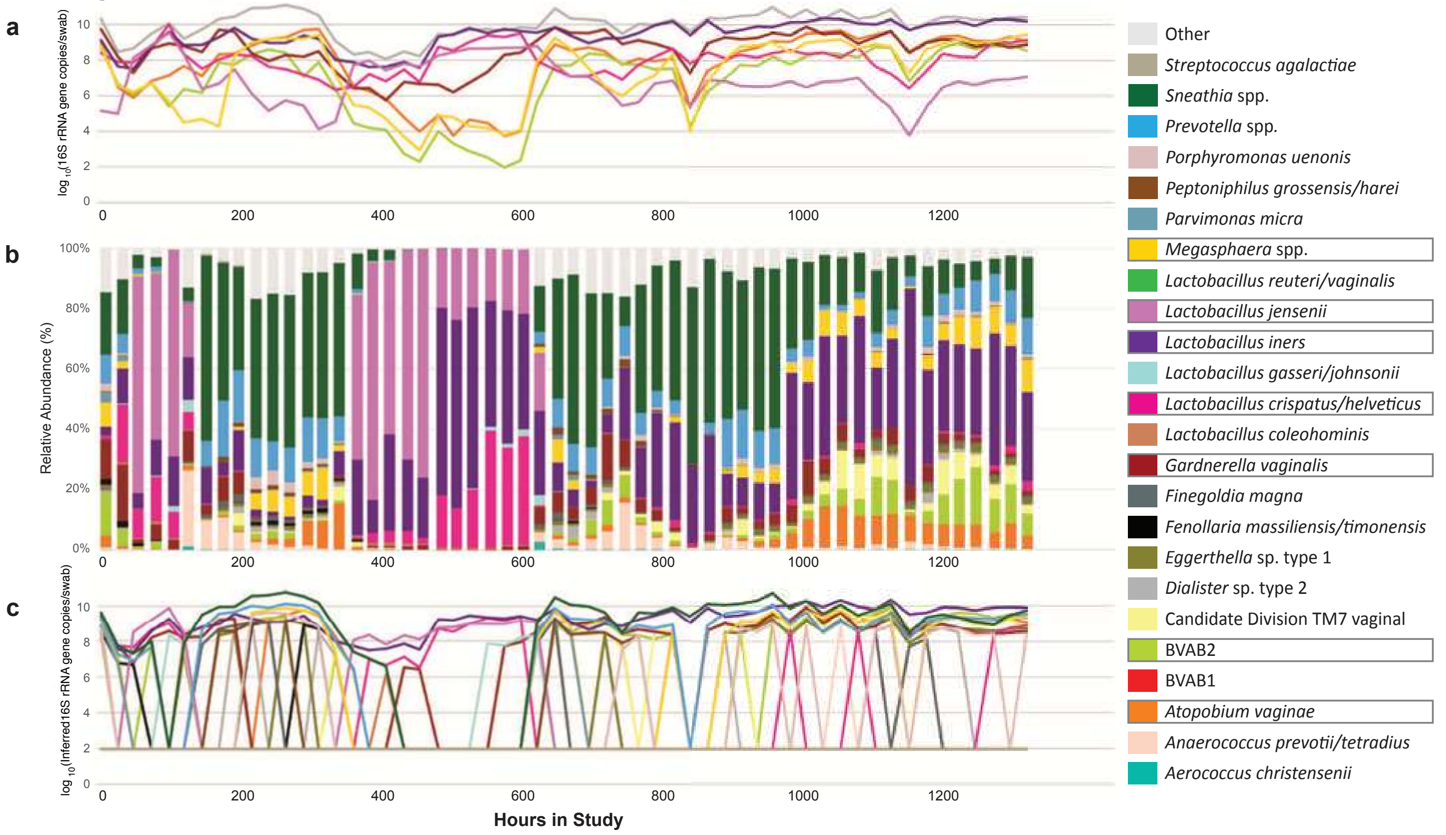

Participant 16

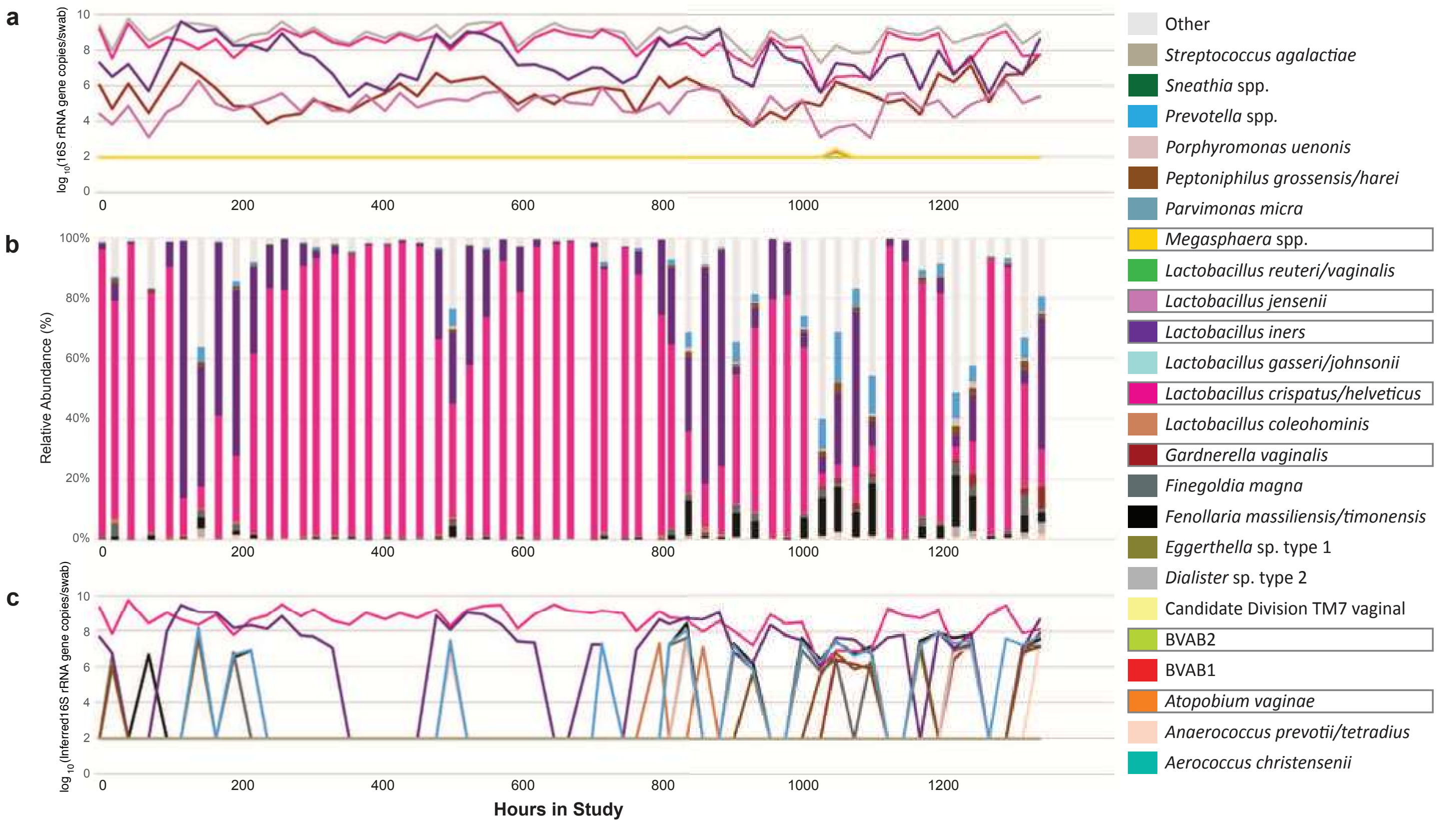

Participant 17

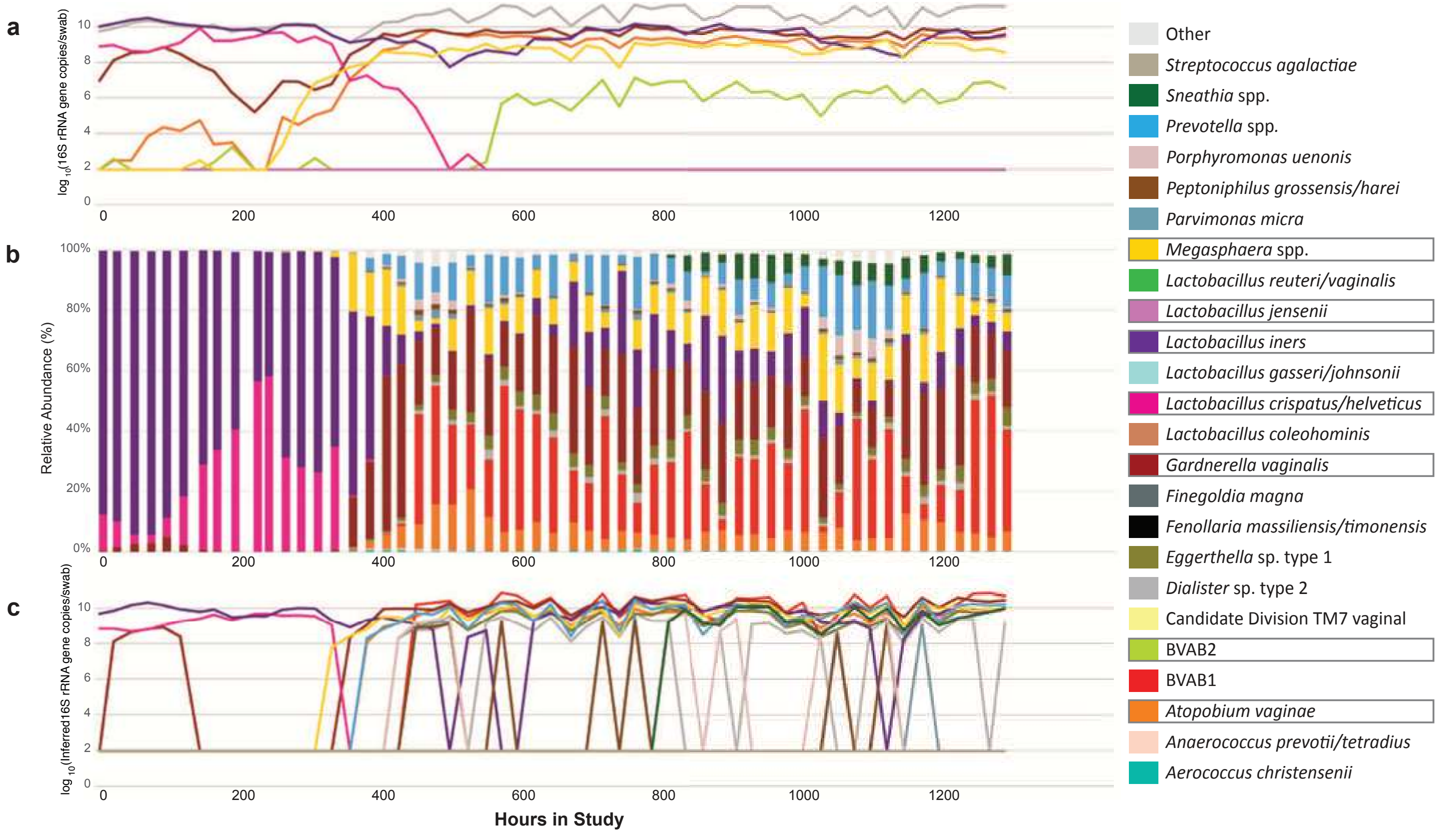

Participant 18

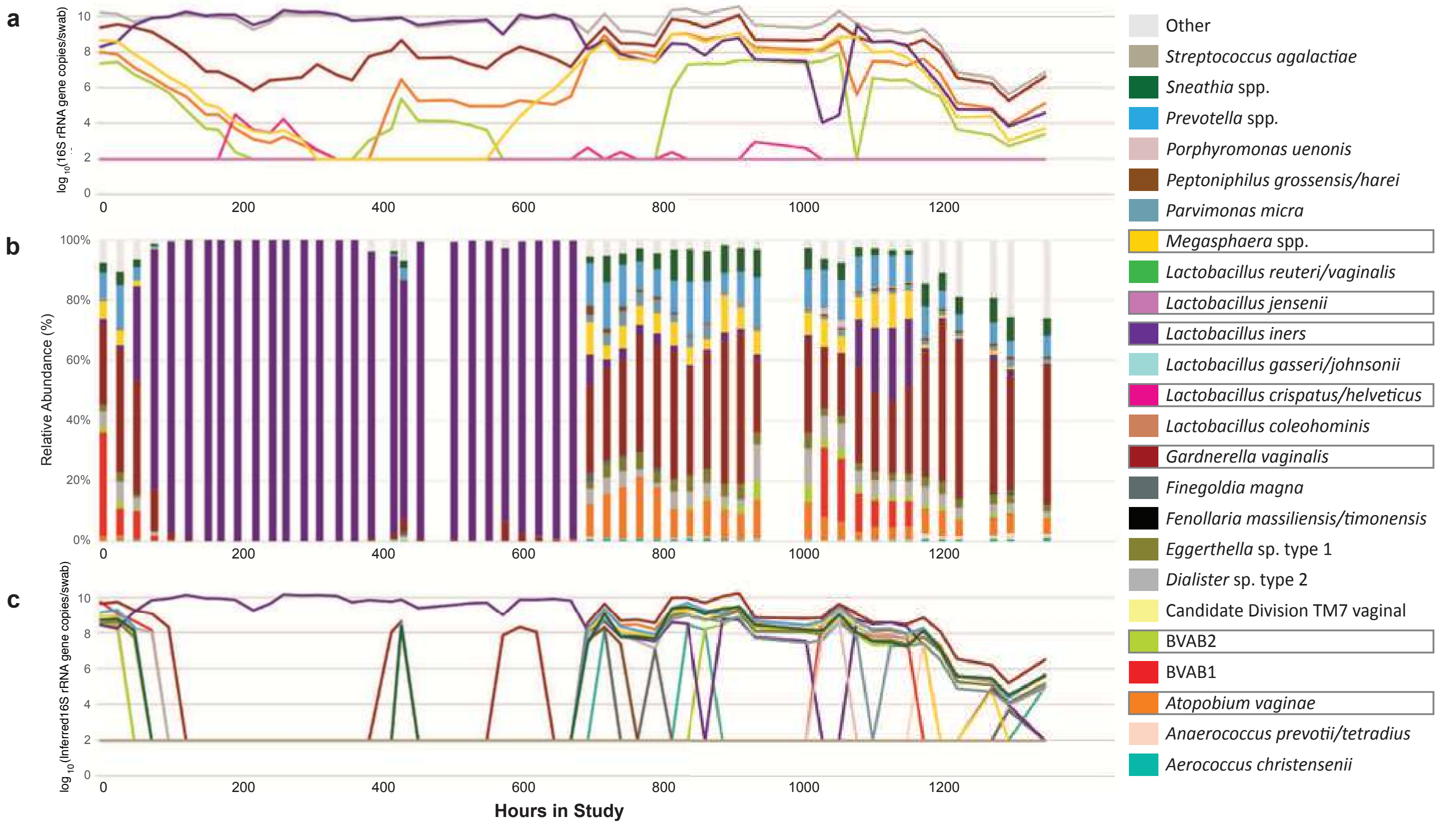

Participant 19

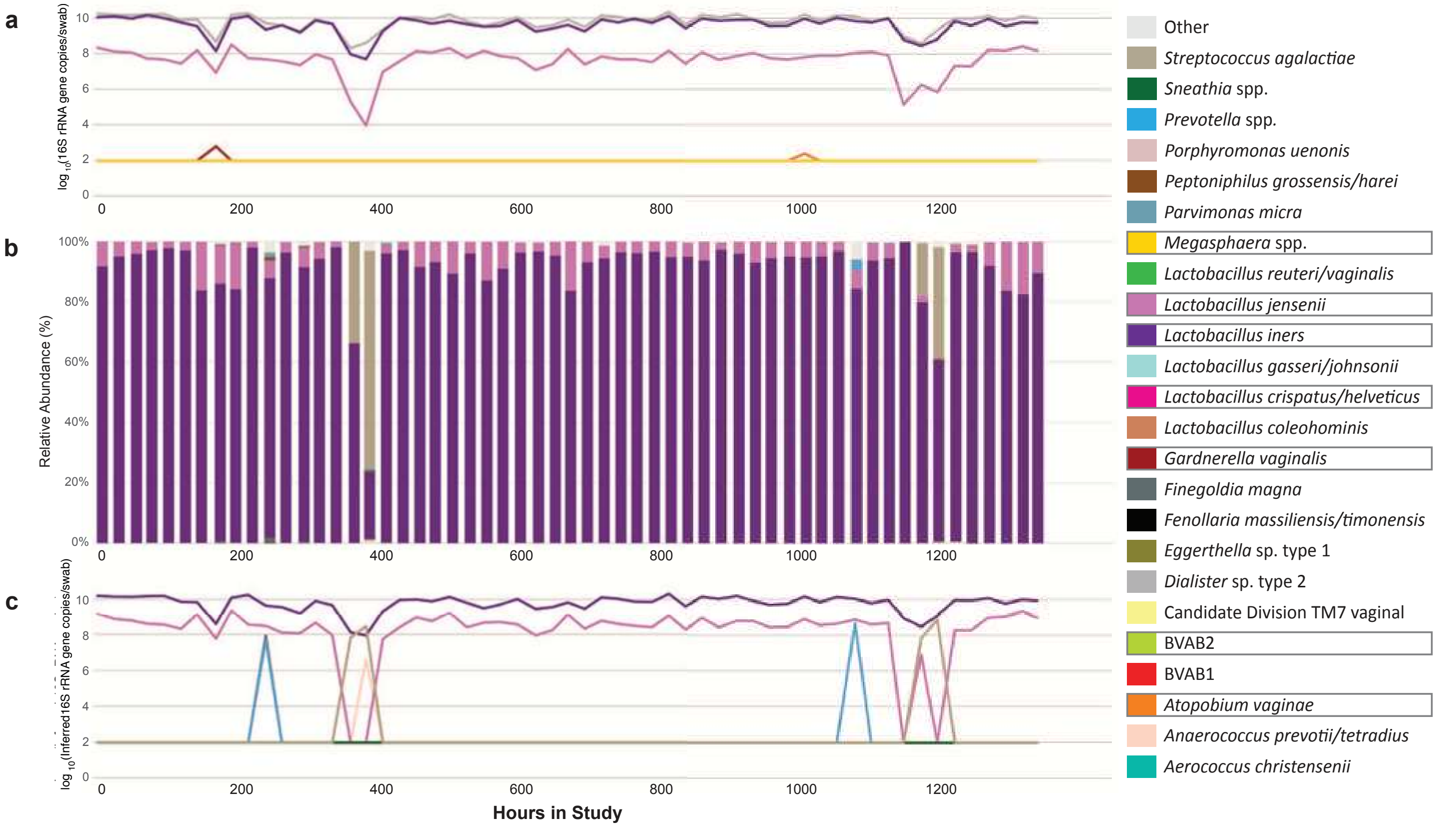

Participant 20

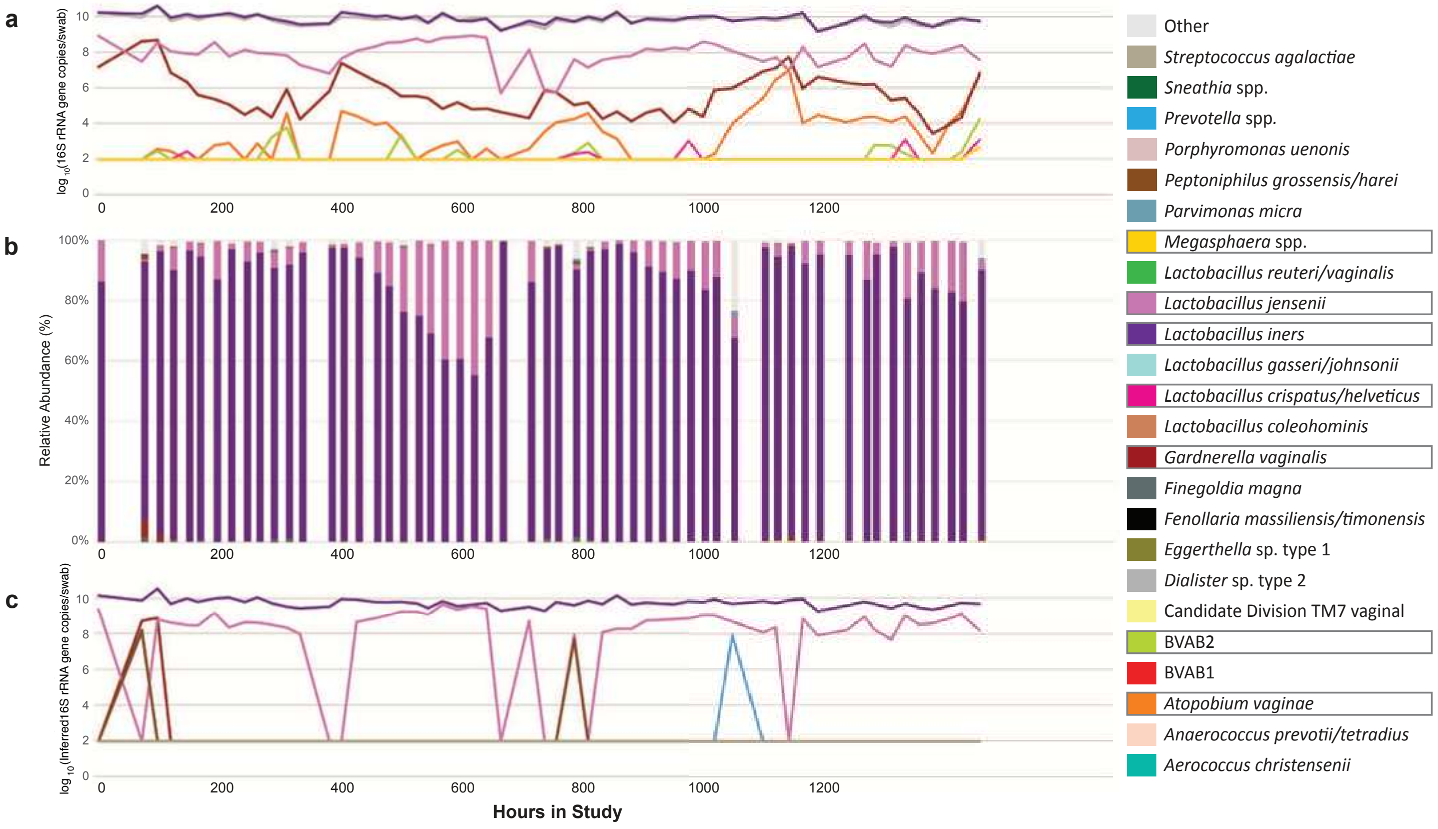

Supplement: FIG S1 [file mSystems.00777-19-sf001.pdf]
