# Supplementary figures and images for: Complementing 16S rRNA Gene Amplicon Sequencing with Total Bacterial Load To Infer Absolute Species Concentrations in the Vaginal Microbiome
Source: mSystems. 2020 Apr 7;5(2):e00777-19. doi: 10.1128/mSystems.00777-19 (PMC7141891; doi:10.1128/mSystems.00777-19)

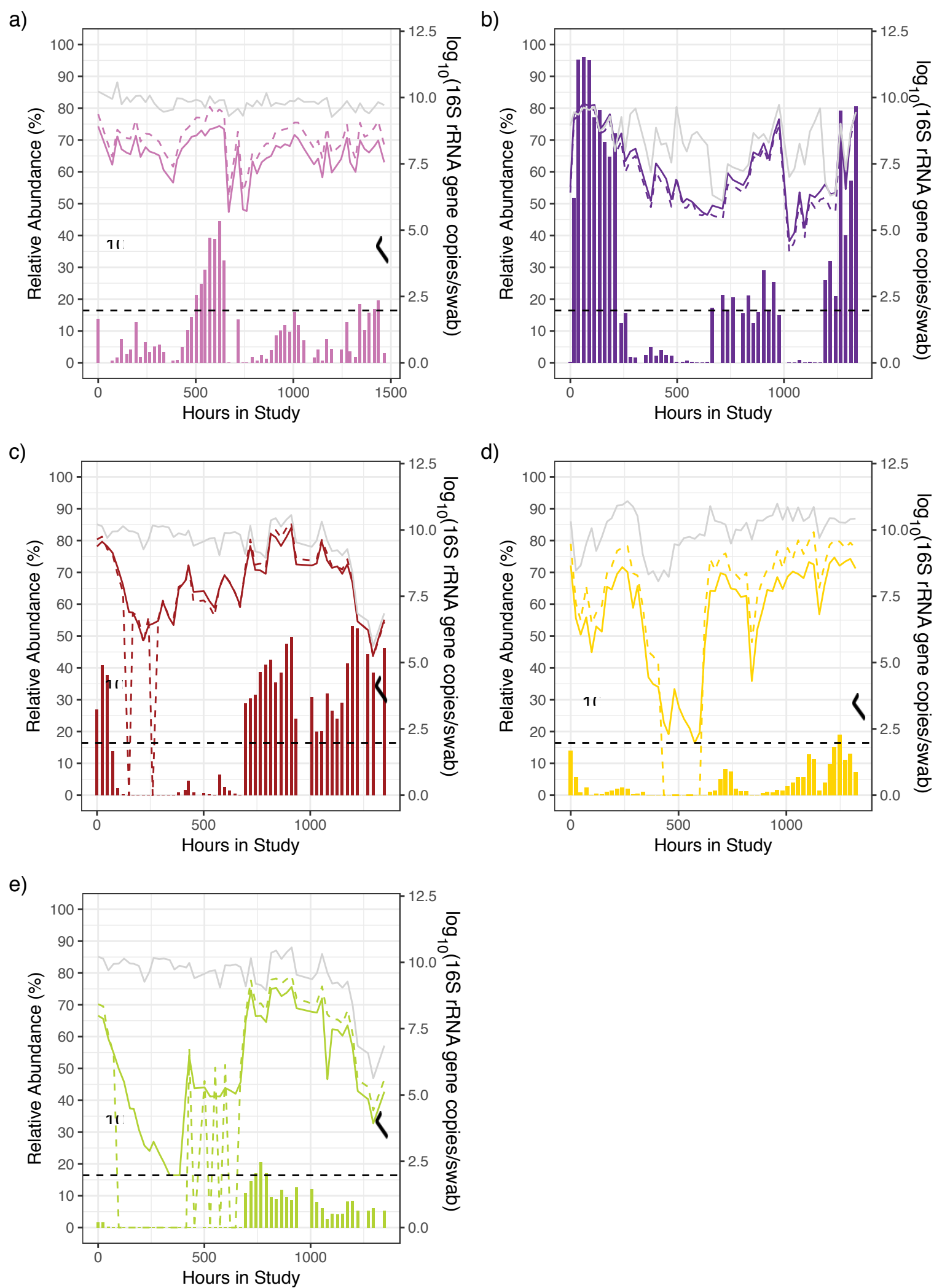

Supplement: FIG S2 [file mSystems.00777-19-sf002.pdf]

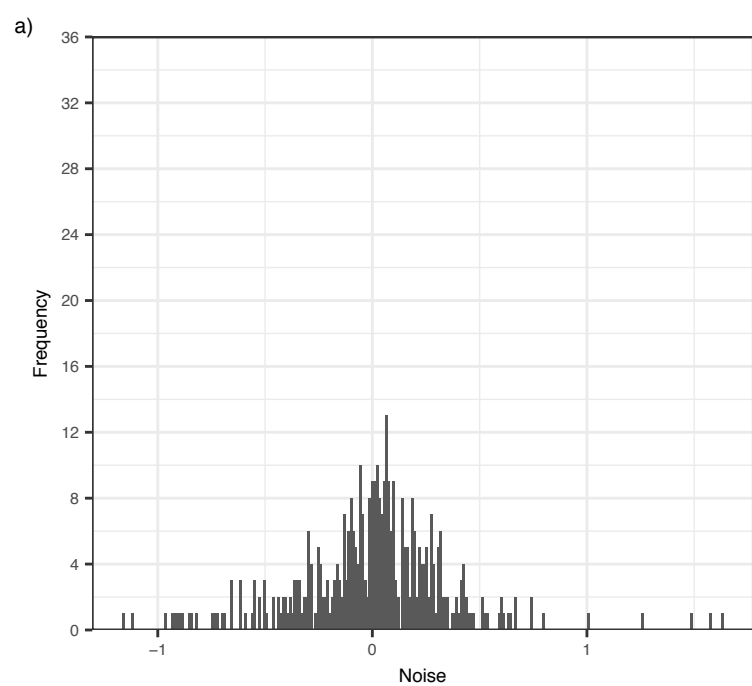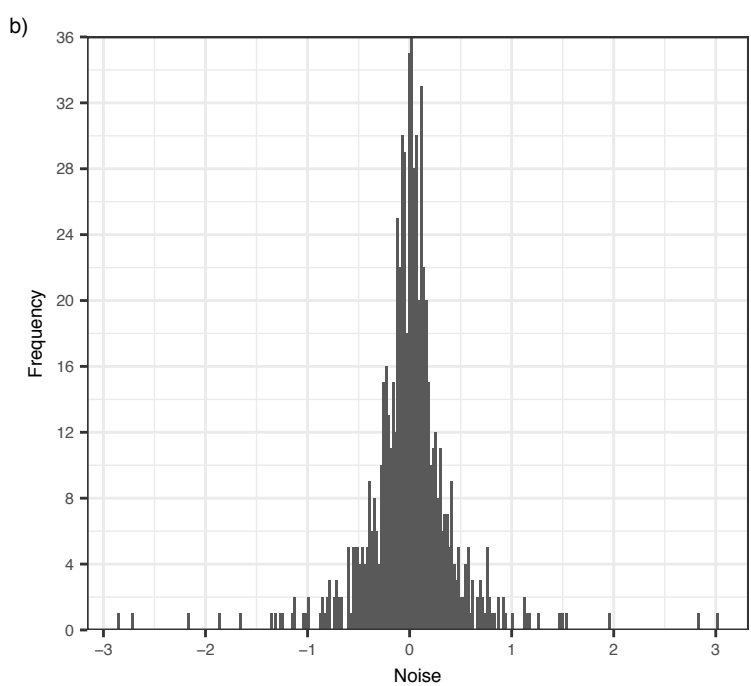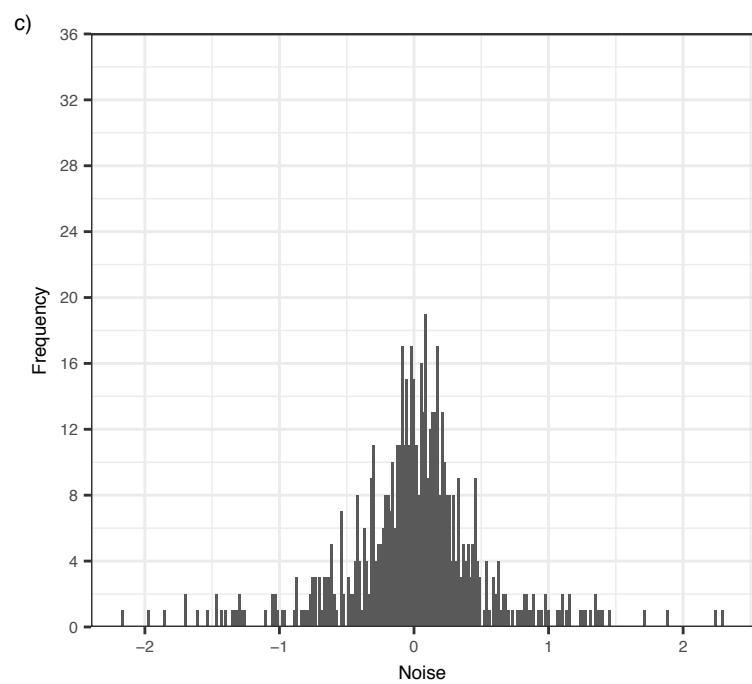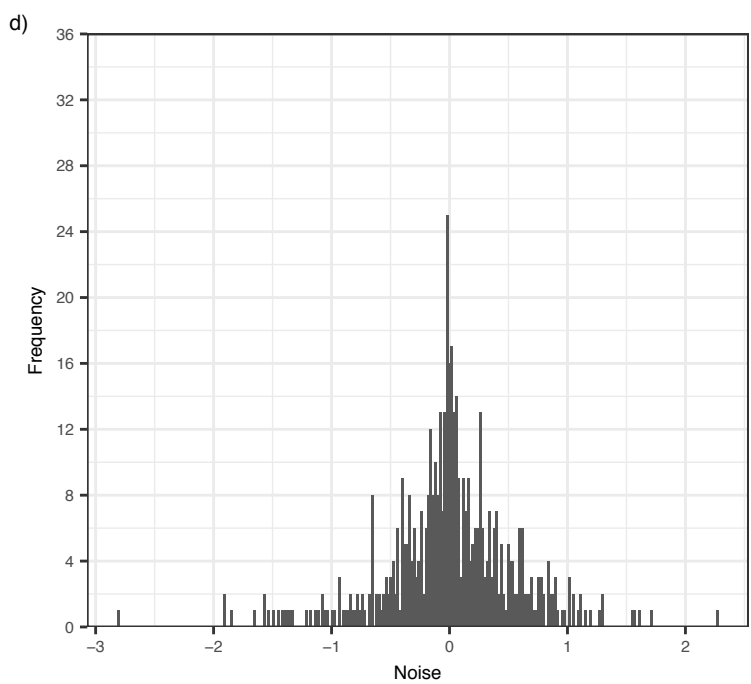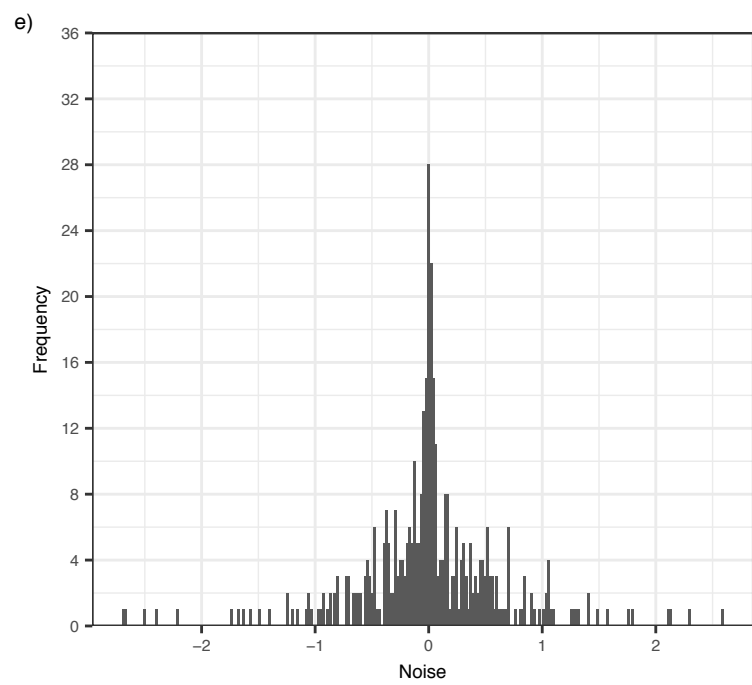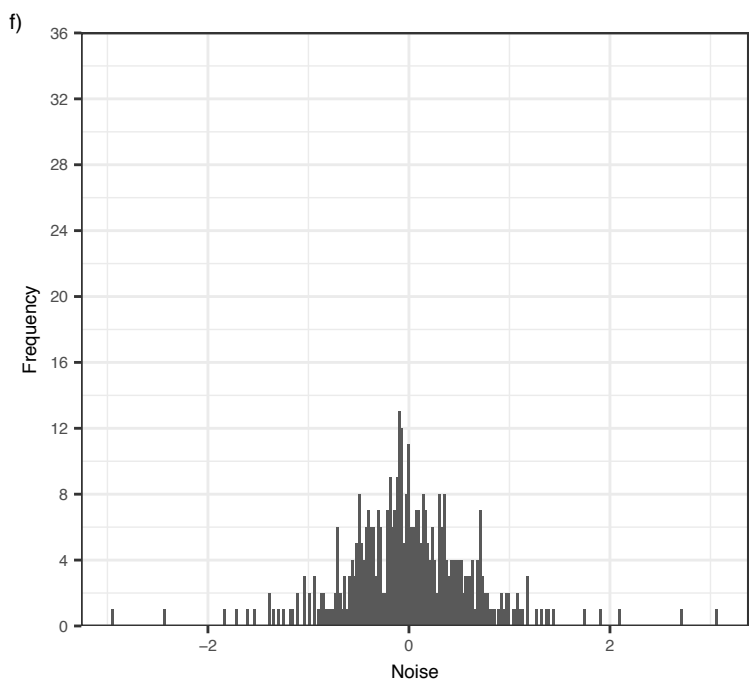

Supplement: FIG S3 [file mSystems.00777-19-sf003.pdf]

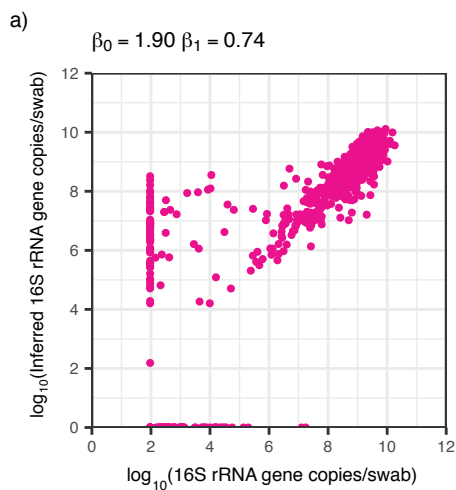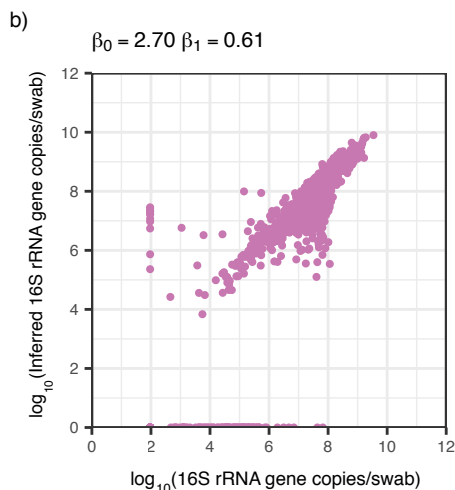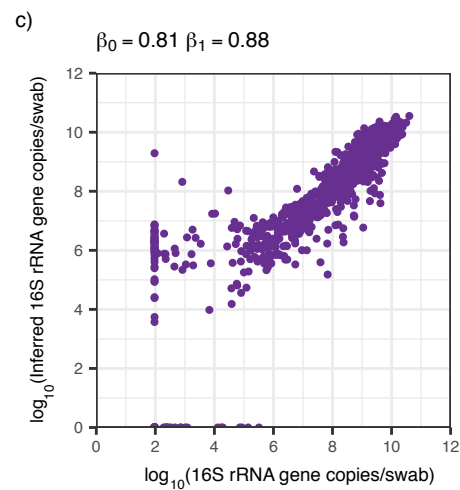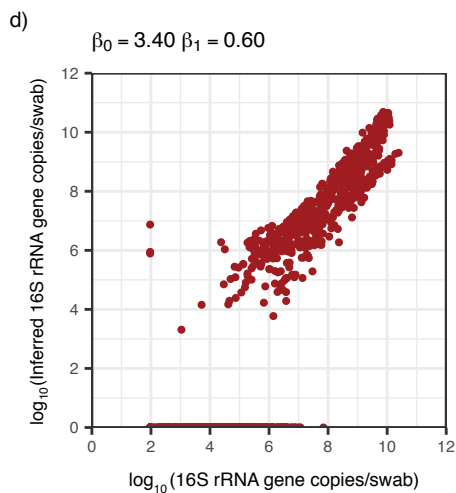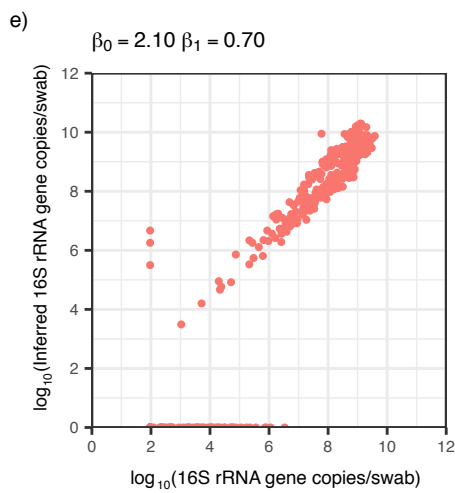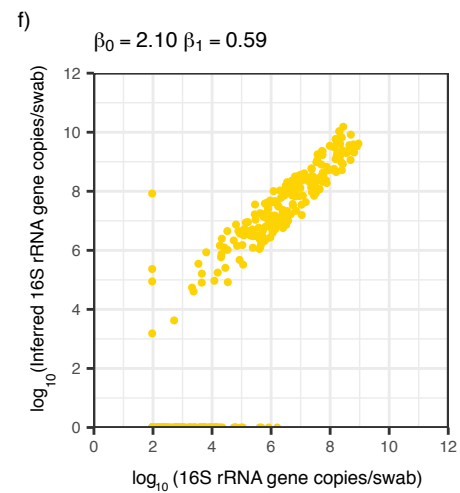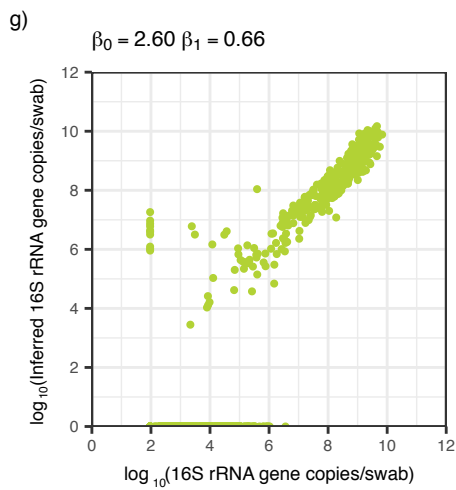

Supplement: FIG S4 [file mSystems.00777-19-sf004.pdf]

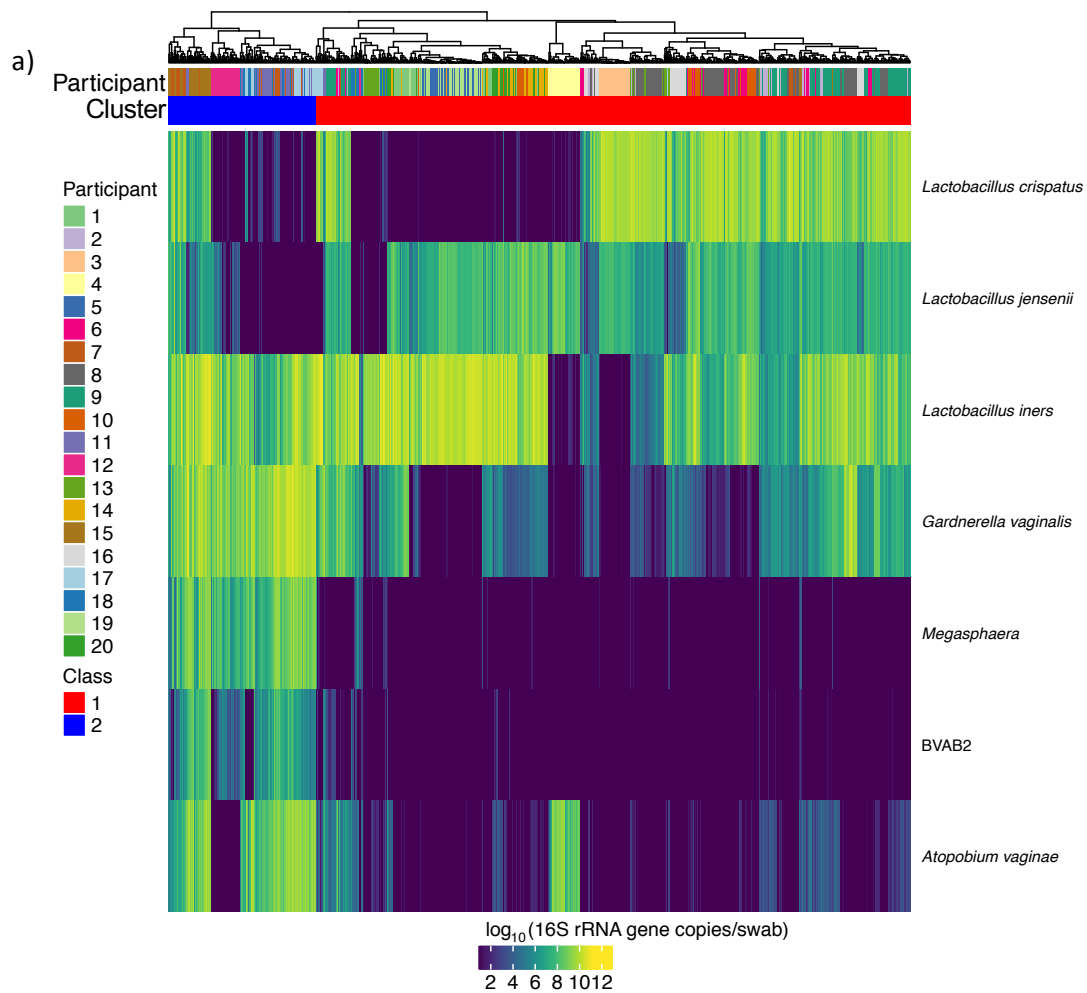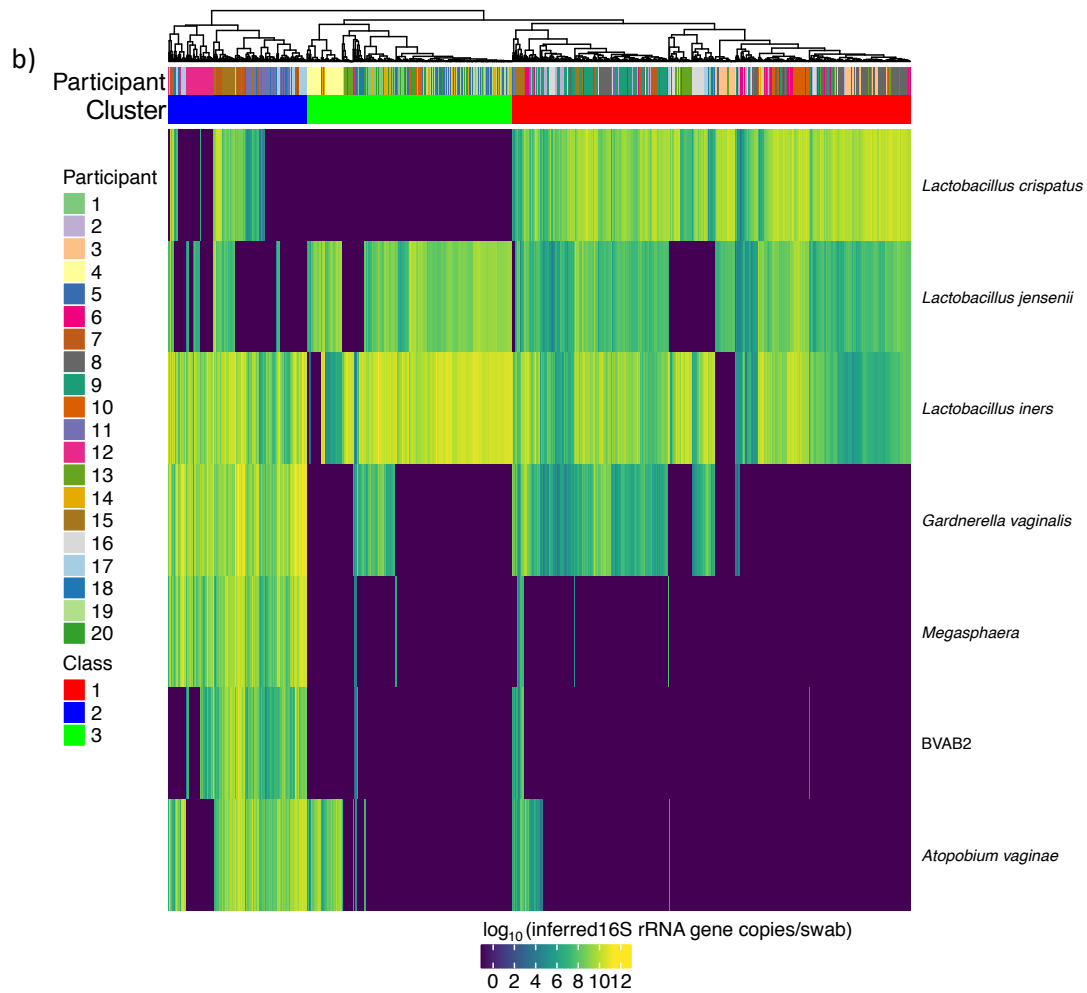

Supplement: FIG S5 [file mSystems.00777-19-sf005.pdf]

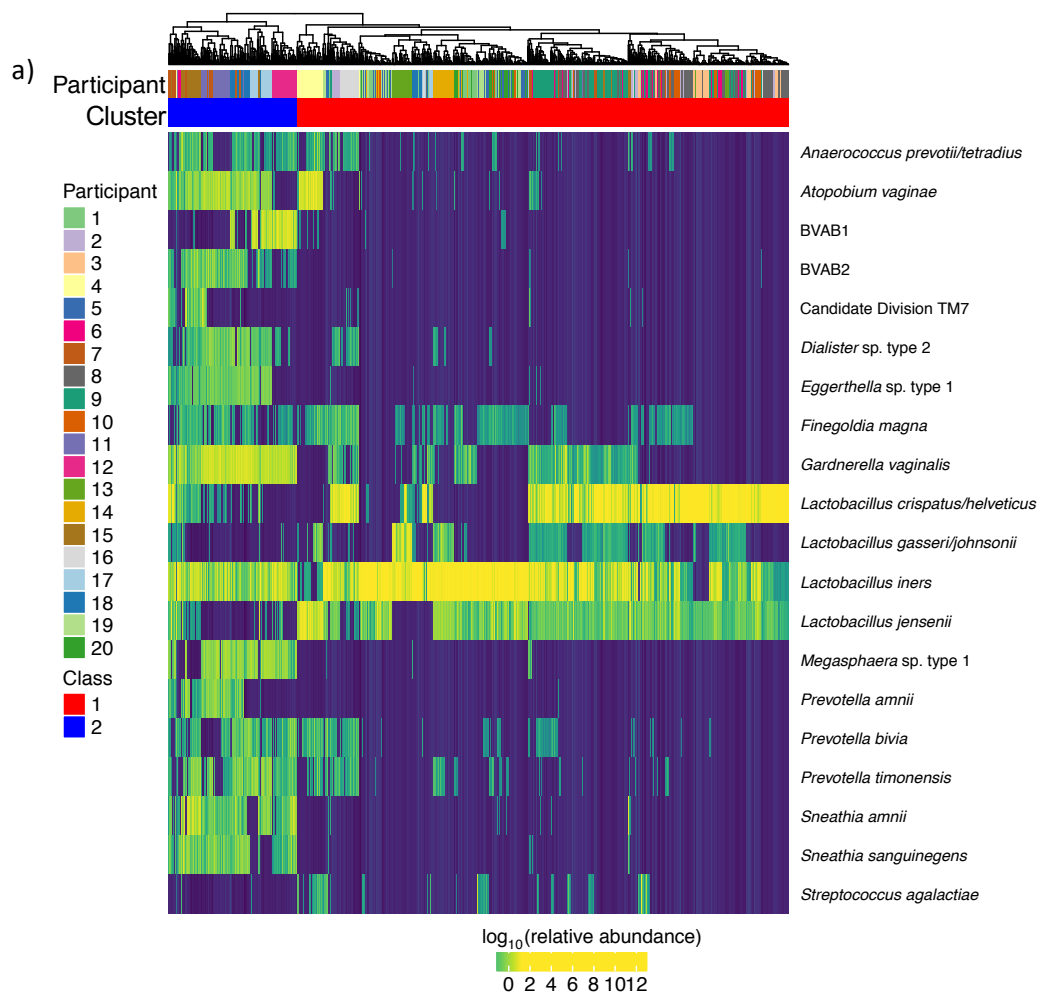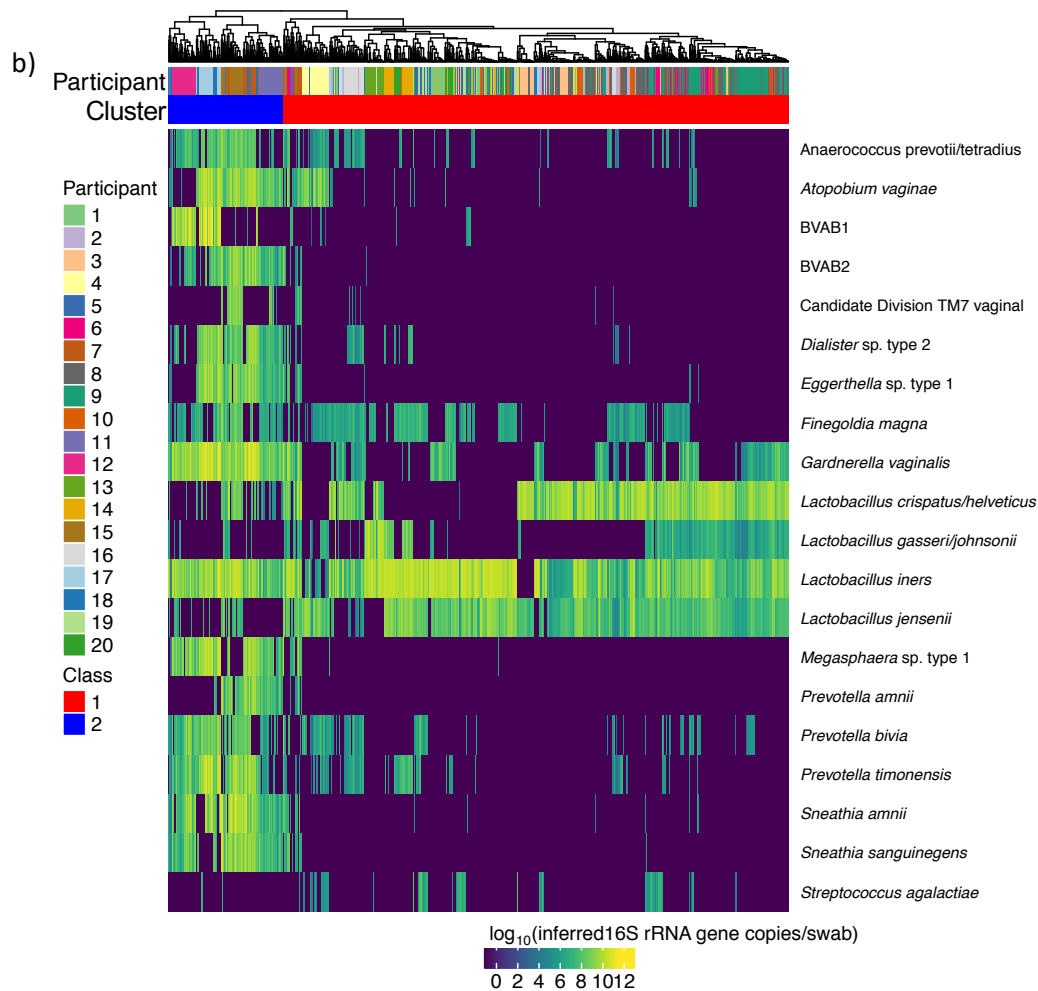

Supplement: FIG S6 [file mSystems.00777-19-sf006.pdf]
